# Glioblastoma upregulates SUMOylation of hnRNP A2/B1 to eliminate the tumor suppressor miR-204-3p, accelerating angiogenesis under hypoxia

**DOI:** 10.1038/s41419-023-05663-w

**Published:** 2023-02-21

**Authors:** Qindong Guo, Yang Fan, Qingtong Wang, Boyan Li, Wei Qiu, Yanhua Qi, Ziwen Pan, Shouji Zhang, Shulin Zhao, Kehui Yang, Hao Xu, Ming Li, Zijie Gao, Jianye Xu, Huizhi Wang, Shaobo Wang, Qilin Tang, Jiawei Qiu, Xing Guo, Lin Deng, Ping Zhang, Rongrong Zhao, Hao Xue, Chengwei Wang, Gang Li

**Affiliations:** 1 Department of Neurosurgery, Qilu Hospital, Cheeloo College of Medicine and Institute of Brain and Brain-Inspired Science, Shandong University, Jinan, Shandong China; 2 Shandong Key Laboratory of Brain Function Remodeling, Jinan, Shandong China; 3 Department of Emergency Medicine, Qilu Hospital, Shandong University, Jinan, China; 4 Department of Neurosurgery, The Affiliated Yantai Yuhuangding Hospital of Qingdao University, Yantai, Shandong China; 5 Department of Neurosurgery, The Affiliated Taian City Central Hospital of Qingdao University, Taian, Shandong China; 6 Department of Neurosurgery, The Second Hospital of Shandong University, Jinan, Shandong 250033 China

**Keywords:** CNS cancer, CNS cancer

## Abstract

Glioma is the most common malignant tumor of the central nervous system in adults. The tumor microenvironment (TME) is related to poor prognosis in glioma patients. Glioma cells could sort miRNA into exosomes to modify TME. And hypoxia played an important role in this sorting process, but the mechanism is not clear yet. Our study was to find miRNAs sorted into glioma exosomes and reveal the sorting process. Sequencing analysis of glioma patients cerebrospinal fluid (CSF) and tissue showed that miR-204-3p tends to be sorted into exosomes. miR-204-3p suppressed glioma proliferation through the CACNA1C/MAPK pathway. hnRNP A2/B1 can accelerate exosome sorting of miR-204-3p by binding a specific sequence. Hypoxia plays an important role in exosome sorting of miR-204-3p. Hypoxia can upregulate miR-204-3p by upregulating the translation factor SOX9. Hypoxia promotes the transfer of hnRNP A2/B1 to the cytoplasm by upregulating SUMOylation of hnRNP A2/B1 to eliminate miR-204-3p. Exosomal miR-204-3p promoted tube formation of vascular endothelial cells through the ATXN1/STAT3 pathway. The SUMOylation inhibitor TAK-981 can inhibit the exosome-sorting process of miR-204-3p to inhibit tumor growth and angiogenesis. This study revealed that glioma cells can eliminate the suppressor miR-204-3p to accelerate angiogenesis under hypoxia by upregulating SUMOylation. The SUMOylation inhibitor TAK-981 could be a potential drug for glioma. This study revealed that glioma cells can eliminate the suppressor miR-204-3p to accelerate angiogenesis under hypoxia by upregulating SUMOylation. The SUMOylation inhibitor TAK-981 could be a potential drug for glioma.

## Introduction

Glioblastoma is the most common primary malignant of adult central nervous system and is resistant to radiotherapy and chemotherapy [1]. The median survival period of glioblastoma patient is approximately 15 months [1, 2]. Many studies had revealed that glioma immunosuppressive microenvironment promoted malignant behavior of glioma, but the formation of glioma immunosuppressive microenvironment and the interactions between glioma cells, immune cells and stromal cells in glioma microenvironment are still not clear [3].

Exosomes are extracellular vesicles with diameters of 30–150 nm [4]. Exosomes can transport cargo, including nucleic acids and proteins, from donor cells to target cells [5]. Exosomes mediate the crosstalk between tumor cells and other cells in many kinds of tumors, such as colon tumors, breast tumors, and GBM [6–8]. Our previous studies showed that GBM cells and non-GBM cells in the TME could interact with each other through exosomes, and the noncoding RNAs (miRNAs, lncRNAs and circRNAs) transported by exosomes played important roles in tumor growth, immune escape and vasculogenic mimicry [9–12]. Recently, some studies found that some noncoding RNAs were selectively loaded rather than randomly packaged into exosomes, and the sorting process was dependent on specific RNA-binding proteins (RBPs) [13, 14].

Small ubiquitin-like modifier (SUMO) is a kind of posttranslational modification. SUMOylation (addition of the SUMO modification) is the process by which SUMO proteins (sumo1, sumo2, sumo3, etc.) conjugate SUMO to a specific lysine residue of the target protein with the assistance of SAE1, SAE2, and UBC9 [15–17]. The deSUMOylation process is mainly regulated by the sentrin-specific protease (SENP) family in mammals [17, 18]. SUMOylation participates in many important biological processes, including protein degradation, DNA repair, proliferation, etc. [19–22]. The members of the heterogeneous nuclear ribonucleoprotein (hnRNP) family are RBPs [23]. Studies have shown that some members of the hnRNP family, such as hnRNP A2/B1, hnRNP Q and hnRNP A1, are related to the exosome-sorting process of noncoding RNA, and SUMOylation plays an important role in this sorting process, but the mechanism is still unclear [14, 24, 25].

To study the sorting mechanism of noncoding RNAs in exosomes and how these noncoding RNAs affect the GBM tumor microenvironment, we collected glioma tissue, normal tissue, and pre- and postsurgery cerebrospinal fluid (CSF) from 47 glioma patients at Qilu Hospital of Shandong University. We observed that miR-204-3p tended to be sorted into exosomes. We found that miR-204-3p suppressed tumors via the CACNA1C/MAPK pathway in GBM cells. We confirmed that hnRNP A2/B1 mediated the exosome sorting of miR-204-3p by pull-down and RIP assays, and this process was dependent on a specific motif and domain. Further research showed that hypoxia could upregulate the expression of the transcription factor SOX9 to promote the synthesis of miR-204-3p. Hypoxia could also upregulate UBC9 expression to promote SUMOylation of hnRNP A2/B1. SUMOylation-mediated cytoplasmic localization of hnRNP A2/B1 promoted exosome sorting of miR-204-3p. miRNA-204-3p transported by exosomes promoted angiogenesis and migration of vascular epithelial cells through the ATXN1/STAT3 pathway. The SUMOylation inhibitor TAK-981 inhibited glioblastoma growth and angiogenesis by arresting SUMOylation of hnRNP A2/B1.

## Results

### miR-204-3p tends to be sorted into exosomes of GBM cells

**Fig. 1:**
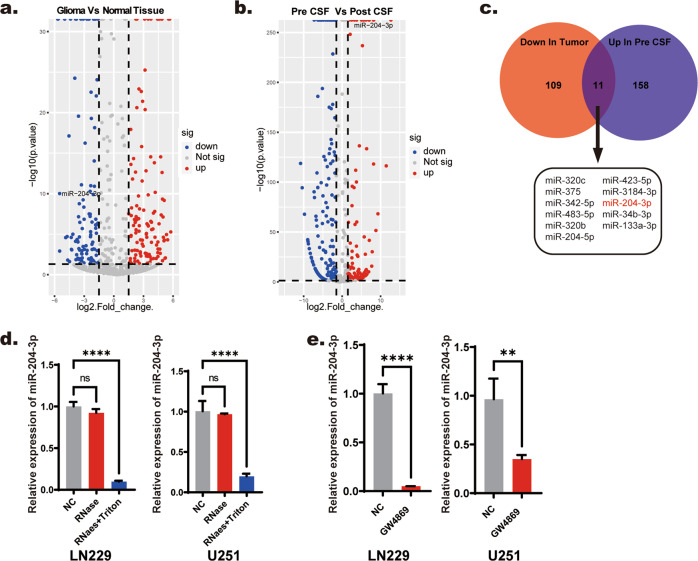
miR-204-3p was tended to be sorted into exosomes of GBM cells. **a** Volcano plots of miRNAs that were downregulated in glioma compared with normal tissue. **b** Volcano plots of miRNAs that were upregulated in CSF exosomes before surgery (pre-CSF) compared with CSF exosomes after surgery (post-CSF). **c** Venn diagram showing miRNAs downregulated in glioma and upregulated in pre-CSF. **d** RNase treatment was used to evaluate the stability of miR-204-3p in the medium supernatant of LN229 and U251. **e** Treatment with exosome inhibitor GW4869 was used to verify that miR-204-3p existed in exosomes.

We first performed whole-transcriptome sequencing of CSF exosomes (before and after surgery) and tumor tissue collected from 44 patients who underwent surgery for GBM at Qilu Hospital of Shandong University from November 2017 to October 2019. We also collected 12 nontumorous brain tissue samples from craniocerebral trauma patients from the Department of Neurosurgery of the Qilu Hospital of Shandong University, the Second Hospital of Shandong University, and the 5th People’s Hospital of Jinan Shandong University as a control group. Three control CSF samples were collected from normal pressure hydrocephalus patients. Exosomes isolated from CSF samples using ultracentrifugation were identified by Western blotting, Zeta View, and transmission electron microscopy (Supplementary Fig. 1a–c). The DESeq2 package in R was used to normalize and identify differentially expressed miRNAs in tissue and CSF exosomes. We found 271 significantly differentially expressed (*P* < 0.05 and absolute value of fold change >1.5) miRNAs in glioma tissue and 433 miRNAs in CSF exosomes. To identify miRNAs with a significant exosome sorting process, we took the intersection of miRNAs upregulated in CSF exosomes before surgery and miRNAs downregulated in glioma tissue and found 11 candidate miRNAs (Fig. 1a–c). MiR-204-3p was selected for further research because of its high fold change and expression level in exosomes (Supplementary Fig. 1d, e). Because CSF exosomes could be secreted from several cell types in the central nervous system, we first wanted to confirm whether miR-204-3p was present in glioma cell exosomes. We found that miR-204-3p was present in the culture media of LN229 and U251 cells and was tolerant to RNase digestion (Fig. 1d). GW4869, a widely used exosome inhibitor, significantly reduced miR-204-3p expression in LN229 and U251 exosomes (Fig. 1e). Exosomes isolated from the GBM cell lines LN229 and U251 using ultracentrifugation were identified by Western blotting, Zeta View, and transmission electron microscopy (Supplementary Fig. 1f–h). These results showed that miR-204-3p tended to be sorted into GBM exosomes.

### miR-204-3p inhibits GBM growth through the CACNA1C/MAPK pathway

**Fig. 2:**
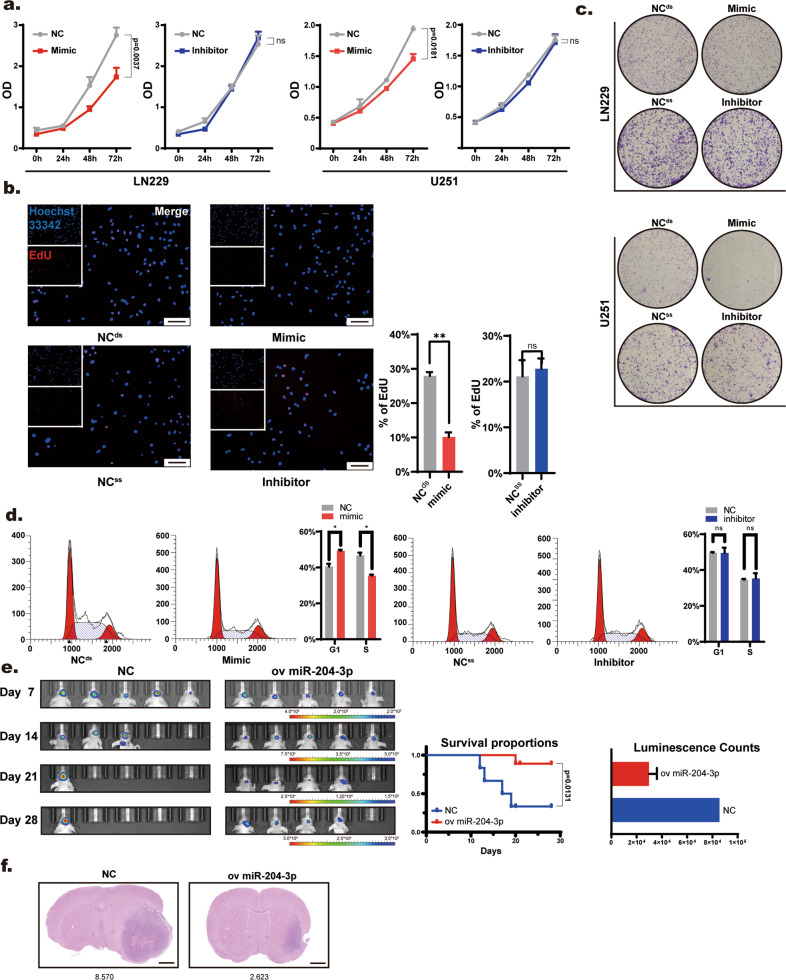
miR-204-3p inhibited GBM growth in in vitro and vivo. **a** CCK8 assay showed that miR-204-3p mimics suppressed proliferation of LN229. **b** Plate cloniny formation assay of LN229 after miR-204-3p mimic and inhibitor transfection. **c** EdU showed 204-3p mimic inhibited the proliferation of LN229 cells. Bar = 100 μm. **d** Flow cytometry showed that mimics of 204-3p caused G1/S arrest in LN229 cells. **e** Live imaging showed that overexpression of miR-204-3p inhibited tumor growth and prolonged survival time in vivo. **f** IHC showed tumor volume was smaller in the miR-204-3p overexpression group. Bar = 1000 μm.

We first studied the function of miR-204-3p in GBM cell lines. miR-204-3p mimics and inhibitor were transfected into LN229 and U251 cells, respectively. The transfection efficiency was confirmed by qRT–PCR (Supplementar Fig. 2a). CCK8, colony-forming assays, and EdU fluorescence staining showed that miR-204-3p mimics inhibited the proliferation of GBM cell lines, while the miR-204-3p inhibitor showed no significant effect (Fig. 2a–d and Supplementar Fig. 2b, c). The ineffectiveness of the miR-204-3p inhibitor may be due to the low expression level of miR-204-3p in GBM cell lines. Flow cytometry showed that miR-204-3p inhibited LN229 and U251 proliferation by arresting the cell cycle (Fig. 2b). To further confirm the function of miR-204-3p in vivo, a miR-204-3p vector and miR-204-3p overexpression LN229 cell line were constructed via lentivirus transfection, and overexpression efficiency was confirmed by qRT–PCR (Supplementar Fig. 2d). The orthotopic murine GBM model showed that the miR-204-3p overexpression group exhibited reduced tumor volume and a longer survival period (Fig. 2e). HE staining showed that tumor volume decreased significantly in miR-204-3p overexpression group (Fig. 2f). And immunohistochemistry of mouse brains showed that the expression of proliferation marker ki67 decreased significantly in overexpression group (Supplementar Fig. 2d).

**Fig. 3:**
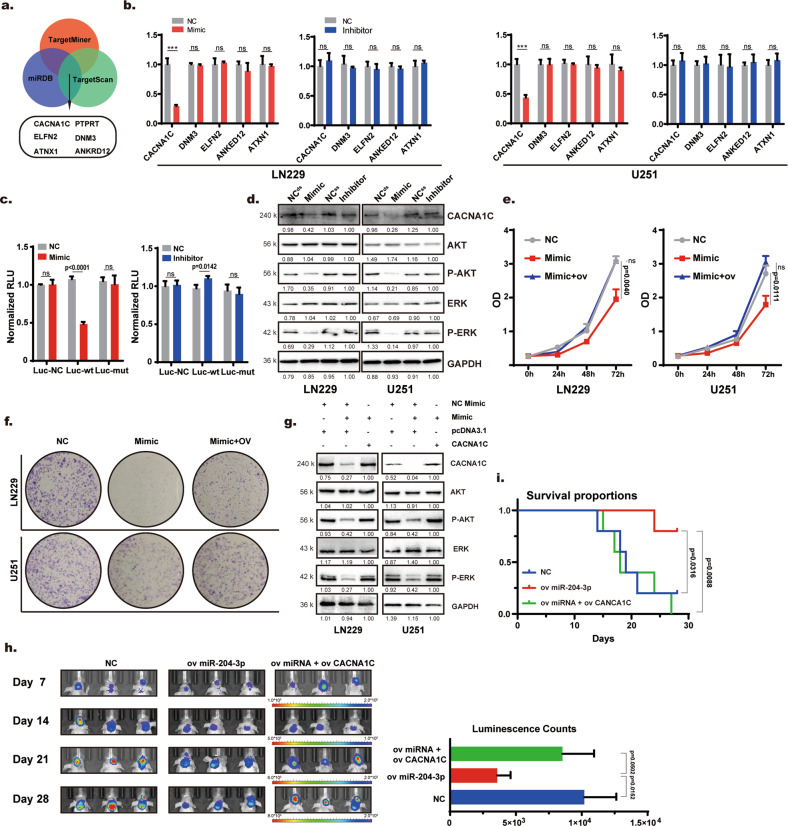
miR-204-3p inhibited GBM proliferation through CACNA1C/MAPK pathway. **a** Venn diagram showing candidate of miR-204-3p targets. **b** qRT-PCR was used to detect the relative expression of candidates of miR-204-3p in LN229 and U251 cells after transfection with mimics and inhibitors of miR-204-3p. **c** Dual luciferase reporter genes were used to verify target gene of miR-204-3p. **d** Western blot analysis showed that 204-3p mimics transfection downregulated CACNAV1C, P-AKT, P-ERK but had no significant influence on either AKT or ERK. **e**–**g** CCK8, plate cloning, and Western blotting showed that overexpression of CACNAV1C rescued the 204-3p transfection induced proliferation inhibition and downregulation of the CACNAV1C/MAPK pathway. **h** Live imaging showed that overexpression of CACNA1C rescued miR-204-3p induced inhibition of tumor growth. **i** Overexpression of CACNA1C reversed the prolonged survival period caused by miR-204-3p.

miRDB, TargetScan, and TarBase were used to predict the target mRNA of miR-204-3p (Fig. 3a). qRT–PCR was used to confirm the content of candidate mRNAs in LN229 and U251 cells after transfection with miR-203-3p mimics and inhibitor, respectively. The results showed that CACNA1C may be a target of miR-204-3p in LN229 and U251 cells (Fig. 3b). Double luciferase reporter gene experiments verified that the target of miR-204-3p in GBM is CACNA1C (Fig. 3c). KEGG pathway analysis showed that CACNA1C is upstream of the MAPK pathway (not shown here). Western blot analysis showed that miR-204-3p mimics downregulated CACNA1C, p-AKT and p-ERK expression, while there was no obvious change in AKT or ERK expression (Fig. 3d). We further constructed CACNA1C-overexpressing cells via lentiviral transfection. CCK8, colony-forming assays showed the antitumor effect of miR-204-3p mimics was rescued by overexpression of CACNA1C (Fig. 3e, f). CACNA1C overexpression also rescued miR-204-3p mimics-induced MAPK pathway inhibition (Fig. 3g). siRNA was used to knock down CACNA1C in GBM cell lines. CCK8, colony-forming, flow cytometry, and Western blot assays showed that knockdown of CACNA1C inhibited the proliferation of LN229 and U251 cells (Supplementary Fig. 3a–d). The orthotopic murine GBM model showed that CACNA1C overexpression rescued the tumor growth inhibition caused by 204-3p overexpression and significantly shortened the survival period. (Fig. 3h, i). These results showed that miR-204-3p inhibited GBM growth through the CACNA1C/MAPK pathway.

### hnRNP A2/B1 mediated exosome sorting of miR-204-3p

**Fig. 4:**
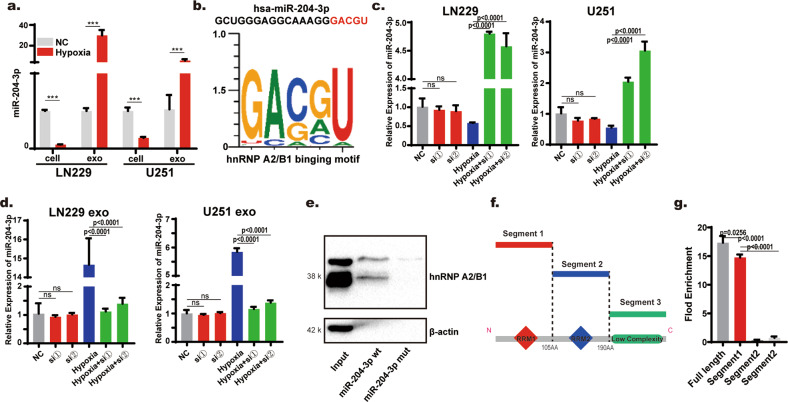
hnRNP A1/B2 mediated exosome-sorting of miR-204-3p. **a** qRT-PCR showed that miR-204-3p was upregulated in LN229 and U251 exosomes but downregulated in LN229 and U251 cells. **b** hnRNP A2/B1 binding motif predicted by starbase. **c**, **d** Knockdown of hnRNP A2/B1 reversed hypoxia mediated exosome sorting of hnRNP A2/B1. **e** RNA pull-down showed that a mutant specific sequence of miR-204-3p canceled the binding between miR-204-3p and hnRNP A2/B1. **f** Structure of Flag-tagged deleted segments of hnRNP A2/B1. **g** RIP assay showed that hnRNP A2/B1 segments without RRM1 could not bind with miR-204-3p.

It has been reported that hypoxia plays an important role in the release of exosomes and affects exosome composition [9, 26]. We found that hypoxia increased the miR-204-3p content in exosomes while reducing the miR-204-3p content in cells (Fig. 4a). The above results verified that miR-204-3p tended to be sorted into exosomes, and this sorting process was influenced by hypoxia. It has been reported that the hnRNP family can mediate the exosome-sorting process of noncoding RNA through a combination of specific RNA sequences. There was an hnRNP A2/B1 binding sequence at the 3′ end of miR-204-3p, as revealed by StarBase, a website widely used to predict the binding motif of RBPs (Fig. 4b). qRT–PCR was used to analyze the content of miR-204-3p in LN229 and U251 cells and exosomes transfected with small interfering RNA of hnRNP A2/B1. The knockdown efficiency was verified by Western blotting (sFig. 4a). We found that knockdown of hnRNP A2/B1 did not significantly change the miR-204-3p content in cells or exosomes under normal oxygen but reversed hypoxia-mediated exosome sorting of miR-204-3p (Fig. 4c, d). To further verify whether hnRNP A2/B1 binding to miR-204-3p with a specific motif, biotin-labeled wild-type and mutant sequences of miRNA-204-3p were designed (Supplementary Fig. 4b). In vitro RNA pull-down assays revealed that the mutant sequence eliminated the interaction between miR-204-3p and hnRNP A2/B1 (Fig. 4e). To further determine whether the specific domain of hnRNP A2/B1 is involved in binding to miR-204-3p, flag-labeled hnRNPA2/B1 deletion segments were constructed according to the hnRNP A2/B1 structure (Fig. 4f). RNA-immunoprecipitation assays showed that miR-204-3p bound with full-length hnRNPA2/B1 and RRM1 motif segments (Fig. 4g). These results showed that hnRNPA2/B1 directly interacted with miR-204-3p and that hnRNPA2/B1 mediated exosome sorting of miR-204-3p in hypoxia. We also found that miR-204-3p content was higher in hypoxia group than that in normoxic group, after eliminated the effect of exosome-sorting process on intracellular miR-204-3p by hnRNP A2/B1 knockdown (Fig. 4c). This result suggested that hypoxia may promoted the miR-204-3p synthesis.

### Hypoxia promoted the localization of hnRNP A2/B1 to the cytoplasm through upregulation of SUMOylation

**Fig. 5:**
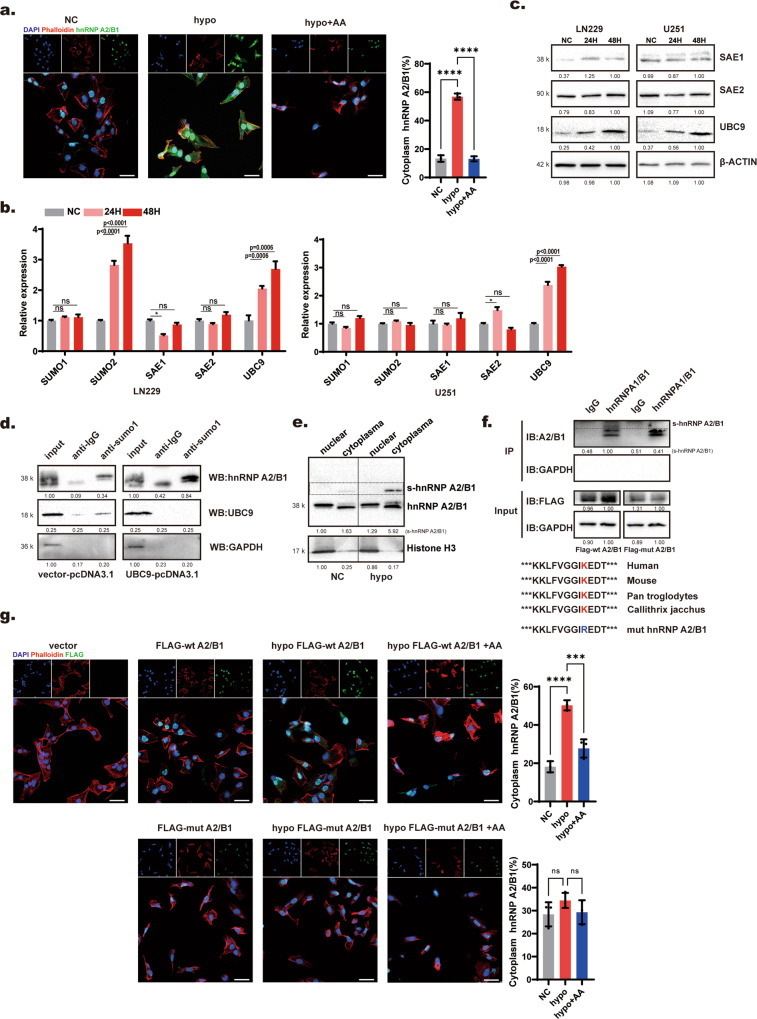
Hypoxia promoted the distribution of hnRNP A2/B1 into the cytoplasm through upregulation of UBC9. **a** Immunofluorescence showed that the proportion of hnRNP A2/B1 increased in the cytoplasm under hypoxia, and this process could be inhibited by the SUMOylation inhibitor anacardic acid (AA) at 100 μM in U251 cells. **b**, **c** qRT-PCR and Western blotting showed that the SUMO-related gene UBC9 was upregulated under hypoxia in LN229 and U251. **d** Immunoprecipitation showed that overexpression of UBC9 increased SUMOylation of hnRNP A2/B1. **e** Western blot analysis showed that SUMOylated hnRNP A2/B1 mainly located in cytoplasm, and upregulated under hypoxia. **f** Immunoprecipitation showed that mutant arginine 108(th) removed the SUMOylation of hnRNP A2/B1. **g** Immunofluorescence showed that mutant hnRNP A2/B1 could not move to the cytoplasm under hypoxia in U251 cells.

It has been reported that the formation of exosomes occurs in the cytoplasm, but hnRNP A2/B1 is mainly located in the nucleus [23, 27]. If hnRNP A2/B1 mediates the packaging of miRNA into exosomes, this packaging process may be influenced by the transport of hnRNP A2/B1 from the nucleus into the cytoplasm. In addition, hypoxia increased hnRNP A2/B1-mediated exosome sorting of miR-204-3p. We hypothesized that hypoxia mediated the localization of hnRNP A2/B1 to the cytoplasm to promote exosome sorting of miR-204-3p. Cellular immunofluorescence staining of LN229 and U251 cells showed that the proportion of hnRNP A2/B1 in the cytoplasm increased under hypoxia (Fig. 5a and Supplementary Fig. 5a). It has been reported that SUMOylation plays an important role in hnRNP A2/B1-mediated exosome sorting of noncoding RNAs, but the mechanism is not clear. Interestingly, we found that AA, an inhibitor of SUMOylation, inhibited hypoxia-mediated cytoplasmic transport of hnRNP A2/B1 (Fig. 5a and Supplementary Fig. 5a). Considering that SUMOylation mediates biological processes, including the nucleocytoplasmic localization of proteins, hypoxia may affect the movement of hnRNP A2/B1 into the cytoplasm through SUMOylation. Hypoxia is one characteristics of glioma tumor microenvironment. In order to confirm the SUMOylation level of hnRNP A2/B1 in glioma tissue, we collect glioblastoma tumor tissue and adjacent normal tissue. Western blot showed that SUMOylation level of hnRNP A2/B1 was increased in tumor tissue (Supplementary Fig. 5b). And the miR-204-3p was downregulated in tumor tissue (Supplementary Fig. 5c). qRT–PCR and Western blotting showed that the SUMOylation-associated gene UBC9 was upregulated under hypoxia (Fig. 5b). To further verify whether upregulation of UBC9 promotes SUMOylation of hnRNP A2/B1, we overexpressed UBC9 with an overexpression plasmid in 293T cells. Immunoprecipitation assays showed that the SUMOylation of hnRNP A2/B1 was enhanced after UBC9 overexpression (Fig. 5d). To confirm whether hypoxia mediated the movement of hnRNP A2/B1 into the cytoplasm, a Paris kit (Thermo Fisher, Cat. AM1921) was used to extract protein from the cytoplasm and nucleus, respectively. Western blot analysis showed that hypoxia upregulated the SUMOylation of hnRNP A2/B1 and that SUMOylated hnRNP A2/B1 was mainly distributed in the cytoplasm (Fig. 5e). Next, we wanted to identify the SUMOylated amino acid residues of hnRNP A2/B1. GPS-SUMO, a software for SUMOylation site prediction, was used to predict the SUMOylation site of hnRNP A2/B1. The results showed that the arginine 108(th) could be the SUMOylation site of hnRNP A2/B1, and the NCBI database showed that this arginine site is conserved (Fig. 5f). We constructed a Flag-labeled wild-type/mutant hnRNP A2/B1 plasmid, and immunoprecipitation showed that mutation of the 108(th) arginine eliminated SUMOylation of hnRNP A2/B1 (Fig. 5f). Cellular immunofluorescence staining showed that the distribution of the SUMOylation site arginine-mutated hnRNP A2/B1 was not influenced by hypoxia (Fig. 5g and Supplementary Fig. 5d). These results showed that hypoxia promoted the movement of hnRNP A2/B1 into the cytoplasm by upregulating SUMOylation of hnRNP A2/B1, and SUMOylation of hnRNP A2/B1 occurred at the 108-arginine residue.

### Hypoxia promoted the transcription of miR-204-3p by upregulating SOX9

**Fig. 6:**
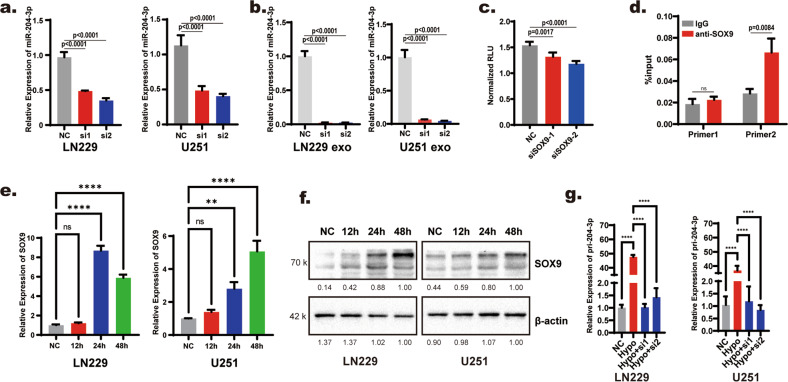
Hypoxia promoted the synthesis of miR-204-3p by upregulating the transcription factor SOX9. **a**, **b** qRT-PCR showed that knockdown SOX9 with small interfering RNA downregulated miR-204-3p in LN229 and U251 cells and exosomes. **c** Dual luciferase reporter genes were used to verify SOX9 binding with promoter region of miR-204-3p. **d** ChIP assay showed that SOX9 bind to the miR-304-3p promoter region. **e**, **f** qRT-PCR and Western blotting showed that SOX9 was upregulated under hypoxia. **g** qRT-PCR showed that knock-down SOX9 inhibited the upregulation of pir-miR-204-3p under hypoxia.

In previous studies, we found that intracellular miR-204-3p levels increased after blocking exosome-sorting progression using small interfering RNAs targeting hnRNP A2/B1 under hypoxic conditions. This result suggested that hypoxia may promote the synthesis of miR-204-3p in hypoxia, but hypoxia promoted the exosome-sorting process to maintain tumor suppression of miR-204-3p at a relatively low level at the same time. qRT–PCR showed that expression of pri-miR-204-3p, the precursor of miR-204-3p, increased under hypoxic conditions (Supplementary Fig. 6a). This result suggested that hypoxia upregulated the transcription of miR-204-3p. The transcription of miRNA is related to its position on the chromosome [28, 29]. miR-204 is in the intron region between exon 1 and exon 2 of host gene, TRPM3. According to previous study, genes in intro regions could been transcript with the host gene [29]. Bioinformatics analysis showed positive correlation between the host gene, TRPM3, and miR-204-3p in glioma patient tumor tissue (Supplementary Fig. 6b). JASPAR predicted that the transcription factor SOX9 binding to the promoter region of TRPM3 (Supplementary Fig. 6c). qRT–PCR showed that siRNA transfection of Sox9 downregulated miR-204-3p expression in both glioblastoma cells and their exosomes (Fig. 6a, b). The knockdown efficiency of si-SOX9 was confirmed by Western blotting (Supplementary Fig. 6d). Double luciferase reporter experiments and chromatin immunoprecipitation (ChIP) verified the interaction of SOX9 and the promoter region of TRPM3 (Fig. 6c, d). Then we wanted to determine whether GBM cells upregulate SOX9 to promote the transcription of miR-204-3p. TCGA database analysis showed that SOX9 is positively related to Hif1α, a hypoxia-related protein, in glioma (Supplementary Fig. 6e). We also found that hypoxia upregulated SOX9 expression at both the mRNA and protein levels in GBM cell lines (Fig. 6e, f). Knockdown of SOX9 reversed the upregulation of miR-204-3p transcription under hypoxic (Fig. 6g). These results showed that hypoxia promoted the transcription of miR-204-3p by upregulating SOX9 in GBM.

### Exosomal miR-204-3p promotes HUVEC angiogenesis through the ATXN1/STAT3 pathway

**Fig. 7:**
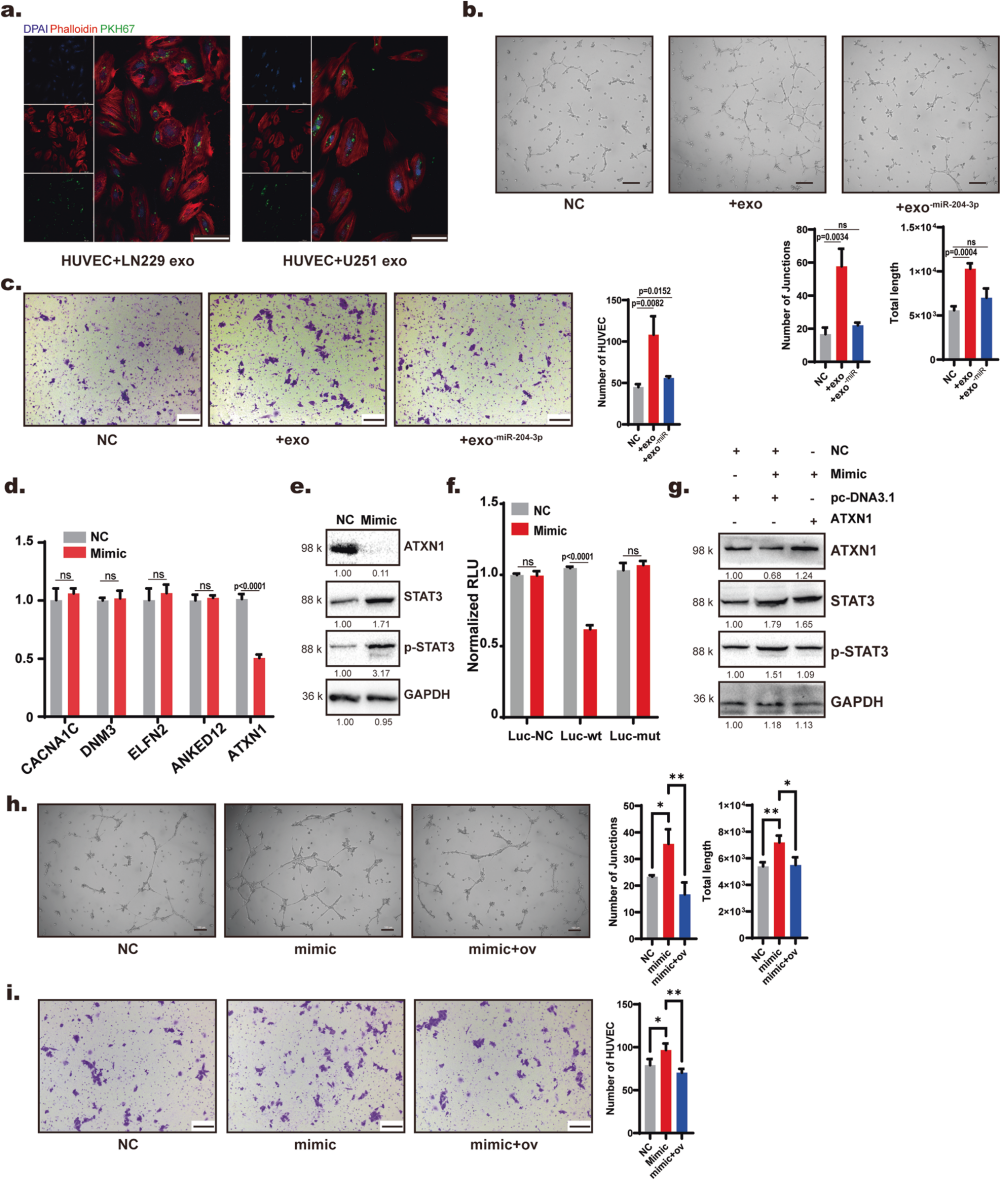
Exosomal miR-204-3p promotes angiogenesis of HUVECs through the ATXN1/STAT3 pathway. **a** Immunofluorescence showed that LN229 and U251 cells could be taken up by HUVECs. Bar = 10 μm. **b**, **c** Exosome treatment promoted angiogenesis and migration of HUVECs. **d** qRT-PCR showed that transfection of miR-204-3p mimics downregulated ATXN1 in HUVECs. **e** Western blot analysis showed that transfection of miR-204-3p mimics downregulated ATXN1 and upregulated both STAT3 and p-STAT3. **f** Dual luciferase reporter genes were used to verify target gene of miR-204-3p in HUVECs. **g**–**i** Overexpression of ATXN1 rescued miR-204-3p promoted angiogenesis, and migration of HUVECs. Bar = 100 μm (**h**, **i**).

Exosomes of glioma cells can be absorbed by several cells in the glioma microenvironment [30–32]. We wanted to determine whether exosomal miR-204-3p influenced the function of cells in the glioma microenvironment. Exosomes have been reported to induce M2-like polarization of tumor-associated macrophages in GBM. We first transfected THP1 cells with mimics and an inhibitor of miR-204-3p. qRT–PCR showed that miR-204-3p mimics did not influence the M1/M2-like phenotype markers of THP1 cells (sFig. 7a). We further examined other cells in the glioma microenvironment. Exosomes and miR-204-3p minus exosomes (exo^-miR^) were separated from LN229 cells and LN229 cells transfected with an inhibitor of miR-204-3p. We found that GBM exosomes were taken up by HUVECs and promote angiogenesis and migration (Fig. 7a, b; scale bar = 10 µm). Exosomes isolated from glioblastoma patient CSF also caused angiogenesis and migration of HUVECs (Supplementary Fig. 7b). Transfection of miR-204-3p mimics also promoted the angiogenesis and migration of HUVECs (Supplementary Fig. 7c). qRT–PCR was used to verify the relative expression of miR-204-3p in HUVECs treated with exosomes (Supplementary Fig. 7d). To identify the target mRNA of miR-204-3p, HUVECs were transfected with mimics of miR-204-3p. qRT–PCR and Western blotting showed that ATXN1, a predicted target of miR-204-3p, was downregulated after mimic transfection (Fig. 7d). miR-204-3p mimics also upregulated both STAT3 and p-STAT3 in HUVECs (Fig. 7e). A double luciferase reporter gene experiment was used for further confirmation (Fig. 7f). Knocking down ATXN1 with siRNA also upregulated both STAT3 and p-STAT3 and promoted the angiogenesis and migration of HUVECs (Supplementary Fig. 7e–g). Overexpression of ATXN1 with the PCDNA3.1 vector in HUVECs rescued both the upregulation of STAT3 and p-STAT3 and promoted angiogenesis and migration, as observed with miR-204-3p mimics (Fig. 7g–i; scale bar = 200 µm). These results showed that exosomes could be taken up by HUVECs, and that exosomal miR-204-3p promoted angiogenesis in HUVECs through the ATXN1/STAT3 pathway.

### The SUMOylation inhibitor TAK-981 suppressed glioblastoma growth and angiogenesis

**Fig. 8:**
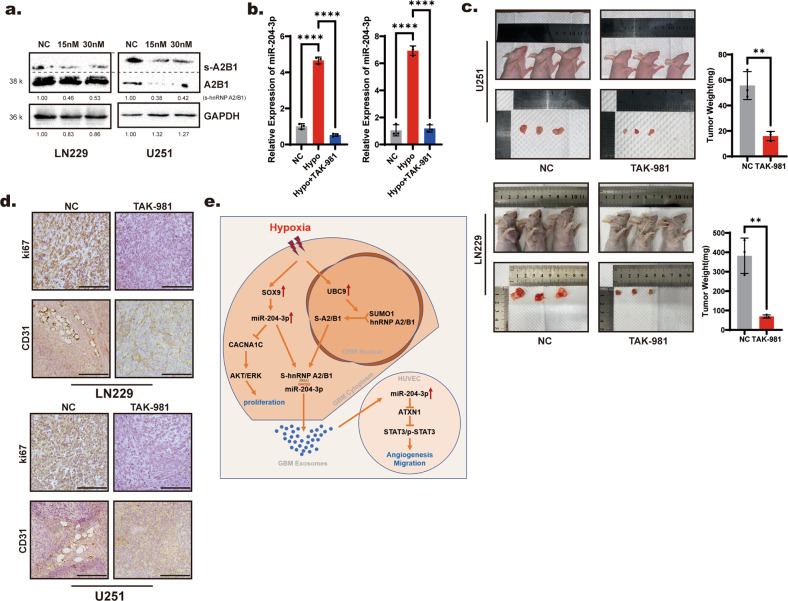
SUMOylation inhibitor TAK-981 suppressed glioblastoma growth and angiogenesis. **a** Western blot showed that TAK-981 downregulated both SUMOylated hnRNP A2/B1 and total hnRNP A2/B1 in LN229 and U251 cells. **b** qRT-PCR showed that TAK-981 downregulated miR-204-3p in exosomes of LN229 and U251 cells under hypoxia. **c** TAK-981 inhibited tumor growth in vivo. **d** IHC showed that TAK-981 inhibited both proliferation marker (Ki67) and vascular epithelial markers (CD31). **e** Pattern diagram.

Considering that the exosome-sorting process of miR-204-3p plays an important role in both the proliferation of glioma and angiogenesis in the tumor microenvironment and that this sorting process is mediated by SUMOylated hnRNP A2/B1, we hypothesized that blocking the SUMOylation of hnRNP A2/B1 is the key to inhibiting exosome sorting of miR-204-3p under hypoxia. TAK-981, a SUMOylation inhibitor, was reported to have potential antitumor effects. In vitro experiments showed that TAK-981 downregulated both SUMOylated hnRNP A2/B1 and total hnRNP A2/B1 expression in glioblastoma cell lines (Fig. 8a). As SUMOylation could counter ubiquitination, the downregulation of total hnRNP A2/B1 may depend on the enhancement of ubiquitination-related degradation. TAK-981 also reduced hypoxia-mediated exosome sorting of miR-204-3p in both LN229 and U251 cells (Fig. 8b). Considering the poor permeability of the TAK-981 blood–brain barrier, a subcutaneous tumor model of glioblastoma in mice was used to study the antitumor effect of TAK-981. The results showed that TAK-981 suppressed the growth of glioblastoma (Fig. 8c). Immunohistochemistry verified that the TAk-981 inhibited not only proliferation but also angiogenesis in glioblastoma (Fig. 8d). These results showed that TAK-981 inhibited glioblastoma growth by inhibiting the SUMOylation of hnRNP A2/B1.

## Discussion

The malignant behavior of GBM is related to its specific tumor microenvironment [33]. Tumor cells and other cells in the GBM tumor microenvironment can interact through various mechanisms, and exosomes play an important role in this process [34]. In addition to other materials, exosomes contain nucleic acids and proteins. In our previous studies, noncoding RNAs in exosomes were shown to play an important role in promoting the formation of the GBM inhibitory immune microenvironment and maintaining the malignant phenotype of GBM cells [9, 12, 32, 35]. Studies have shown that the process by which noncoding RNAs enter exosomes is not completely random, and some noncoding RNAs depend on specific mechanisms to enter exosomes. Noncoding RNAs with shorter sequences, such as miRNAs, are more easily sorted into exosomes [36]. To study this sorting mechanism, cerebrospinal fluid exosomes and glioma tumor tissues were collected from patients, and data analysis showed that miR-204-3p tended to be sorted into exosomes. miR-204-3p inhibits tumor proliferation through the CACNA1C/MAPK pathway. We found that hypoxia played an important role in the transfer of miR-204-3p to exosomes. In a hypoxic environment, the upregulated transcription factor SOX9 in the tumor increased the synthesis of miR-204-3p, but the total content of miR-204-3p in tumor cells was decreased. Hypoxia increased the SUMOylation of hnRNP A2/B1 by upregulating UBC9 expression. SUMOylation promoted the transfer of hnRNP A2/B1 to the cytoplasm, and hnRNP A2/B1 in the cytoplasm promoted the transfer of miR-204-3p to exosomes, ultimately leading to a decrease in miR-204-3p levels in cells. miR-204-3p in exosomes promoted tube formation of vascular endothelial cells through the ATXN1/STAT3 pathway. The SUMOylation inhibitor TAK-981 inhibited the exosome-sorting process of miR-204-3p under hypoxia by blocking SUMOylation of hnRNP A2/B1 to inhibit tumor growth and angiogenesis. The above process is shown with a pattern diagram (Fig. 8e). This study revealed a new mechanism of hypoxia-induced exosome sorting, which provides a new direction for the development of drugs to treat GBM.

Hypoxia can induce malignant behavior in many tumors, but extreme hypoxia often leads to tumor cell death. The upregulation of miR-204-3p synthesis induced by hypoxia may be a normal physiological process under hypoxic pressure to regulate cell growth. However, GBM cells discharged excessive miR-204-3p into exosomes and maintained a relatively low level of tumor suppression of miR-204-3p in cells. miR-204-3p in exosomes promoted angiogenesis of vascular endothelial cells in tumors, which alleviated stress caused by hypoxia. This may be a mechanism by which tumor cells the hypoxic environment. Other cargo in hypoxia-induced tumor exosomes may function via similar mechanisms, which may become a promising focus for exosome-related studies.

SUMOylation-induced localization of hnRNP A2/B1 to the cytoplasm may be related to the nuclear pore structure. It was reported that hypoxia also affected nuclear pore opening [37]. Whether hypoxia also influences the distribution of hnRNP A2/B1 in the cytoplasm by opening the nuclear pore is still not clear and warrants further study. The formation of exosomes is a complex process. How hnRNP family members participate in this process and whether the SUMOylation of hnRNP molecules affects this process are also worthy of further study.

## Conclusion

In conclusion, we found an exosome-sorting miRNA, miR-204-3p, that was downregulated in human glioma tissues and upregulated in human glioma exosomes. miR-204-3p inhibited GBM cell proliferation through the CACNA1C/MAPK pathway. hnRNP A2/B1 mediated the exosome-sorting process of miR-204-3p. Hypoxia upregulated the synthesis of miR-204-3p by upregulating SOX9. Hypoxia also promoted exosome-sorting process of miR-204-3p by upregulating SUMOylation of hnRNP A2/B1. Finally, miR-204-3p was downregulated in GBM cells under hypoxia. miR-204-3p in exosomes was transmitted to the vascular epithelium and promoted angiogenesis through the ATXN1/STAT3 pathway. The SUMOylation inhibitor TAK-981 suppressed glioma proliferation of glioma. Our findings suggest that GBM upregulates SUMOylation of hnRNP A2/B1 to eliminate the tumor suppressor miR-204-3p, accelerating angiogenesis under hypoxia.

## Methods

### Ethics

Experiments with animals were conducted under the guidelines of Qilu Hospital. To obtain human CSF and tumor tissue, samples were collected from glioma patients under a protocol approved by Qilu Hospital after written informed consent was obtained. This study was approved by the Ethics Committee on Scientific Research of Shandong University Qilu Hospital (approval number: KYLL-2018-324).

### Cells

LN229, U251, HUVEC, and 293T cells were purchased from ATCC. Human GBM cell lines (LN229 and U251) and 293T cells were cultured with DMEM supplemented with 10% FBS, penicillin (100 U/ml), and streptomycin (100 μg/ml). HUVECs were cultured with ECM supplemented with 5% FBS, penicillin (100 U/ml), streptomycin (100 μg/ml), and 1% ECGS.

All cells were cultured in a humidified incubator containing 5% CO_2_ at 37 °C. The hypoxia treatment group was cultured in a humidified incubator containing 5% CO_2_ and 1% O_2_ at 37 °C.

### Exosome extraction and storage

Normal FBS in culture medium was replaced with FBS^-exo^ (FBS minus exosomes) 48 h before exosome extraction. The precipitate was removed by supercentrifugation (100,000 × *g*, 12 h) of FBS. First, culture medium was centrifuged at 300 × *g* for 10 min, 2000 × *g* for 10 min, and 10,000 × *g* for 30 min, and the precipitate was removed. Then, the supernatant was centrifuged at 10,000 × *g* for 70 min, and the precipitate was dissolved in PBS. All exosome samples were stored at −80 °C if not used immediately.

### RNase treatment

LN229 and U251 cells were cultured in DMEM with 10% FBS^-exo^. The supernatant was incubated with 5 U/μg RNase R (Epicenter Technologies, USA) or RNase R and 0.1% Triton X-100 for 10 min at 37 °C. The stability of miR-204-3p was analyzed by qRT–PCR.

### HUVEC tube formation assay and migration assay

For tube formation assay, 96-well plates were coated with 70 μl Matrigel (BD, lot: 356234), per well, and incubated for 3 h at 37 °C. HUVECs (5000) were plated into 96-well plates 48 h after transfection or cocultured with exosomes. A microscope (Lecia, DMi8) was used to capture images. Images were analyzed with ImageJ using the Angiogenesis Analyze function.

For migration assay, HUVECs were cultured with serum-free ECM medium in the upper chamber of 24-well transwell (Corning, 3422) without Matrigel coating, and 500 μl complete ECM medium was added to the bottom chamber. After 24 h, migrated HUVECs was fixed in 4% paraformaldehyde for 15 min and stained with 0.1% crystal violet solution (Sigma-Aldrich, HT90132). Five random fields of adherent cells were photographed with a light microscope (Leica, DM2500).

### Immunoblotting and qRT–PCR

Immunoblotting and qRT–PCR were performed following a published protocol [38]. The antibodies used in immunoblotting are listed in Table 1, and the primers used in this study are listed in Table 2. Full and uncropped Western blots were included in Supplementary Material.

**Table 1:** Antibodies.

Name	Company	Cat
anti-CACNA1C	santa	sc-398433
anti-akt	proteintech	60203-2-Ig
anti-pakt	proteintech	80455-1-RR
anti-erk	proteintech	11257-1-AP
anti-perk	proteintech	80031-1-RR
anti-hnRNP A2/B1	santa	sc-53531
anti-GAPDH	proteintech	10494-1-ap
anti-beta-actin	proteintech	20536-1-AP
anti-SAE1	santa	sc-271592
anti-SAE2	santa	sc-376305
anti-UBC9	santa	sc-271057
anti-sumo1	abcam	ab11672
anti-histone	abcam	ab1791
anti-SOX9	abcam	ab185230
anti-ATXN1	santa	sc-365343
anti-stat3	abcam	ab68153
anti-pstat3	abcam	ab76315
anti-calnexin	abcam	ab133615
anti-TSG101	abcam	ab216447
anti-CD9	system biosciences	exoAB-cd9

**Table 2:** Primers.

Homo-Sumo1-94F GTCAAAGACAGGGTGTTCCAA
Homo-Sumo1-94R TCCATTCCCAGTTCTTTTGGAGT
Homo-sumo2-76F GATGGTTCTGTGGTGCAGTT
Homo-sumo2-76R GTCGTTCACAATAGGCTTTCATT
Homo-sumo3-73F AGGGCTTGTCAATGAGGCAGA
Homo-sumo3-73R TGTGCTGGAGTGTCAGTTTCA
Homo-UBC9-70F GGCCTACACGATTTACTGCC
Homo-UBC9-70R AAACTTCTTGGCTTGTGCTCG
Homo-SENP1-176F GGCAAGGACATTTGGACCGA
Homo-SENP1-176R AGATGAGCTTGACGATGGGG
Homo-SENP2-111F CATTCCAGCTGACCACAAAGC
Homo-SENP2-111R AACCTGTCAGTTCACAAGATGA
Homo-SENP3-75F GGCATACCCCCAGCTTACTC
Homo-SENP3-75R CTTGAGTCGGGGTTTGGGAG
Homo-ATXN1-105F CGACTCCAGCACCGTAGAGA
Homo-ATXN1-105R AAAACTTCAACGCTGACCTGGG
homo GAPDH-138F GCACCGTCAAGGCTGAGAAC
homo GAPDH-138R TGGTGAAGACGCCAGTGGA
Homo-CACNA1C-103F ATGCCGTAGGAAGGGACTGG
Homo-CACNA1C-103R AACTCTCCGCTAAGCACACC
Homo-ELFN2-81F CGCAGCACATCAATAGCACC
Homo-ELFN2-81R GGTTGAGCGAGGAGTAGAGC
Homo-ANKRD12-113F TAAACATGGGGAGCGTCCAG
Homo-ANKRD12-113R CCTCTTCGGAATCTGTGTAACTT
Homo-DNM3-149F TGCAGGACGCGTTTTCGG
Homo-DNM3-149R TGTTACAATGCCCGACCCT
Hsa-mir-204-3pF GATGGCTGGGAAGGCAAAG
Hsa-mir-204-3pR TATGGTTGTTCACGACTGGTTCAC
U6-F CAGCACATATACTAAAATTGGAACG
U6-R ACGAATTTGCGTGTCATCC
Homo-SOX9-R AGCGAACGCACATCAAGAC
Homo-SOX9-F CTGTAGGCGATCTGTTGGGG
CHIP primer1 SOX9-F CATCCAGGTTAAAACACAGGCTT
CHIP primer1 SOX9-R CCAAATACCAACTAGGGGGTAGAAT
CHIP primer2 SOX9-F AACCAGACGGCCCTTTTCA
CHIP primer1 SOX9-R GCAGTTGGGGGCATCACAAAT

### Gene silencing and overexpression

Small interfering RNAs (siRNAs), miR-204-3p mimics and inhibitors were synthesized (GenePharma; Shanghai, China). Cell lines were transfected with Lipofectamine™ 2000 reagent (Thermo Fisher Scientific; USA) according to the manufacturer’s protocol. Stable overexpression of CACNA1C and UBC9 in cells was achieved via transfection of the pcDNA3.1-CACNA1C and pcDNA3.1-UBC9 plasmids (GenePharma; Shanghai, China), respectively. Cell lines were transfected with Lipofectamine™ 3000 reagent (Thermo Fisher Scientific; USA) according to the manufacturer’s protocol. Stable overexpression of miR-204-3p was achieved via lentiviral transduction of ov-miR-204-3p (GenePharma; Shanghai, China), with empty vector as a control. All knockdown and overexpression efficiencies were assessed 48 h after transfection by immunoblotting and qRT–PCR. The siRNA and mimic inhibitor sequences are listed below: ATXN1: #1 GGGAATAGGTTTACACAAA and #2 GGTCTAATGTAGGCAAGTA; UBC9: #1 GAGGAAAGCAUGGAGGAAAUU and #2 CCAUCUUAGAGGAGGACAAUU; CACNA1C: #1 GGAAAGCUCUAUACCUGUU and #2 GCUGCACAUUGCCCUUCUUTT; hnRNP A2/B1: #1 GCUGUUAUUGGUGGCUUATT and #2GCUGUUUGUUGGCGGAAUUTT; SOX9: #1 GGAGUUGUGUGCAGAGGAAGC and #2 CGCUCAGGUCAGACUGCAAUA; negative control: UUCUCCGAACGUGUCACGUTT.

### 5-Ethynyl-2′-deoxyuridine (EdU), cell counting kit (CCK)-8 assay and colony formation

An EdU cell proliferation assay kit (RiboBio, #C10310–1; China)) was used to measure GBM cell proliferation rates. Cells were stained according to the manufacturer’s protocol. An inverted fluorescence microscope (Leica, DMi8) was used to capture images.

The proliferation rate was assessed using the ratio of pro-EdU-positive cells to total cells. CCK-8 (Dojindo, Japan) was used to measure cell proliferation according to the manufacturer’s protocol. A microplate reader (Bio-Rad) was used to collect absorbance data at a wavelength of 450 nm.

Cells were cultured in 6-well plates at 2000 cells/well for colony formation. The cell culture medium was changed every 72 h. Cells were fixed with 4% paraformaldehyde for 30 min and stained with crystal violet for 20 min at room temperature after 15 days. Colonies were imaged and quantified with a microscope (Leica).

### Luciferase reporter assays

Firefly luciferase reporters were transfected into 293T cells using Lipofectamine™ 3000 reagent (Thermo Fisher Scientific; USA) according to the manufacturer’s protocol. The reporter genes CACNA1C-wt, CACNA1C-mut, ATXN1-wt and ATXN1-mut were synthesized by GeneChem (Shanghai, China). A Dual Luciferase Reporter Assay Kit (Promega) was used to perform the luciferase assay 48 h later. Luciferase reporter activity was normalized to Renilla luciferase activity.

### Coimmunoprecipitation

IP buffer (Pierce, Rockford, USA) was used to lyse cells 48 h after transfection. Samples were incubated with primary antibody (10 μg antibody per 1000 μg protein) or equivalent IgG overnight at 4 °C. The immunoprecipitated complexes were then incubated with protein A/G agarose beads (Pierce, Rockford, USA) at 37 °C and eluted according to the manufacturer’s protocol. Western blotting was used to detect target proteins.

### RNA pull-down assays

Biotinylated miR-204-3p and its mutated sequence were purchased from RiboBio (GenePharma, Shanghai, China). 293T cells were transfected with biotinylated miR-204-3p and its mutated sequence using Lipofectamine™ 2000 reagent (Thermo Fisher Scientific; USA) according to the manufacturer’s protocol. Then, the cells were lysed and incubated with a biotin probe. Cell lysates were incubated with streptavidin-conjugated agarose magnetic beads (Thermo Fisher Scientific, Waltham, MA, USA) at room temperature. Western blotting was used to detect hnRNP A2/B1 content.

### RNA immunoprecipitation (RIP)

Flag-tagged full-length and partial hnRNP A2/B1 plasmids were synthesized by RiboBio (GenePharma, Shanghai, China). The plasmids were transfected into 293T cells using Lipofectamine™ 3000 reagent (Thermo Fisher Scientific; USA) according to the manufacturer’s protocol. 293T cells were lysed with RIP lysis with RNase inhibitors after 48 h of transfection. Then, cell lysates were incubated with beads coated with IgG or anti-Flag antibodies (Millipore) at 4 °C overnight. An RNeasy MinElute Cleanup Kit (Qiagen, Valencia, CA, USA) was used to extract RNA, and qRT–PCR was used to detect miR-204-3p.

### Immunofluorescence (IF)

LN229 and U251 cells were seeded in 8 wells and transfected with plasmids using Lipofectamine™ 3000 reagent (Thermo Fisher Scientific; USA) according to the manufacturer’s protocol, and the control groups were only treated with Lipofectamine™ 3000 reagent (Thermo Fisher Scientific; USA). Cells were fixed with 4% paraformaldehyde for 15 min and incubated with 0.1% Triton X-100 for 10 min. Then, the cells were incubated with 5% BSA for 30 min at room temperature and incubated with primary antibody at 4 °C overnight. After 3 washes with PBS, the cells were incubated with fluorescence secondary antibody (DyLight 488, Thermo Fisher) for 1 h and then stained with 1 U/mL phalloidin (CA1670, Solarbio) for 20 min. A LeicaSP8 confocal microscope (Lecia Microsystems, Wetzlar, Germany) was used to capture images, and ImageJ was used to analyze the data.

### Animal study

Four- to six-week-old male BALB/c-Nude mice (#D00521) were purchased from GemPharmatech (Wilmington, USA). Mice were housed in Shandong University Animal Facility with a 12-light/12-dark light cycle and temperatures of 20–23 °C with 40–60% humidity.

For miR-204-3p overexpression experiment, mice were randomly assigned into NC (*n* = 5) and ov miR-204-3p (*n* = 5). For CACNA1C-related living image rescue experiments, mice were randomly assigned into NC (*n* = 3), ov miR-204-3p (*n* = 3) and ov miRNA & ov CACNA1C (*n* = 3). For CACNA1C-related survival rescue experiments, mice were randomly assigned into NC (*n* = 5), ov miR-204-3p (*n* = 5) and ov miRNA & ov CACNA1C (*n* = 5). Mice were anesthetized and stereotactically injected with 1 × 10^5^ glioma cells expressing a firefly luciferase gene into the right frontal lobe of the brain (2 mm lateral and 1 mm anterior to the bregma at a depth of 3 mm). Live images were taken at 7, 14, 21, and 28 days after injection.

For the subcutaneously implanted glioma model, mice were randomly assigned into NC (*n* = 3) and TKA-981 (*n* = 3) group. Mice were stereotactically injected with 2 × 10^6^ glioma cells (LN229 or U251) into the right axilla after they were anesthetized. An iPhone XR (Apple, A2108) was used to take photographs at 7, 14, and 21 days after injection. TAK-981 (MCE, HY-111789) was diluted according to the instructions of MCE, and the experimental group was injected 25 mg/kg TAK-981 (2.5 mg/ml) every 3 days after injection of glioma cells.

### Immunohistochemistry assay (IHC)

Brains or subcutaneous tumors isolated from the experimental mice were fixed with 4% paraformaldehyde for 24 h and then dehydrated in gradient sucrose solution. After paraffin embedding, tissues were cut into 4 μm thick sections. The sections were blocked with 10% goat serum and then incubated with antibodies (anti-Ki67, Cell Signaling, 9449S; anti-CACNA1C, Santa Cruz, sc-398433; anti-CD31, Cell Signaling Tech, 3528 S) at 4 °C overnight. Then, standard protocols with horseradish peroxidase-conjugated secondary antibodies and 3′-diaminobenzidine (DAB) were used. After hematoxylin staining, sections were mounted and scanned using a microscope (Lecia DM 2500).

### Flow cytometry

For the cell cycle assay in GBM cell lines, LN229 and U251 cells stained with PI/RNase (BD Biosciences, 550825) were used for flow cytometry. LN229 and U251 cells were harvested by trypsinization and centrifugation at 400 × *g* for 5 min 48 h after the corresponding treatment and stained according to the manufacturer’s instructions. Flow cytometry data were collected using a C6 flow cytometer (BD Biosciences). ModFit LT was used to analyze the cell cycle.

### Chromatin immunoprecipitation (ChIP)

For the ChIP assay, a ChIP Assay Kit (Beyotime, China) was used to precipitate DNA combined with SOX9 according to the manufacturer’s protocol. A PCR Clean Up Kit (Beyotime, China) was used to purify the DNA precipitate. qRT–PCR was used to detect target DNA sequences. Primers were purchased from GenePharma (Shanghai, China). Primer set 1 specify “TATACCCCTTTGTAAGCAACT” region of the promoter of TRPM3, and primer set 2 specify “GTAAAGCCATTGTTTTCAGGT” region of the promoter of TRPM3. The sequences of ChIP primer sets were listed in Table 2.

### Statistical analysis

GraphPad software 9 (GraphPad Software Inc., CA, USA) was used to perform statistical analyses. Student’s t test was used to analyze comparisons between two groups. The Wilcoxon test and one-way ANOVA were used for nonparametric and parametric data. We statistically compared the similar variances between the groups as well. The significant of differences are marked in the figures: *P* > 0.05 not significant (ns), *P* < 0.05 statistically significant (*P < 0.05, **P < 0.01, ***P < 0.001, ****P < 0.0001).

## Supplementary information

Supplementary Figures

Western Blot

Reproducibility Checklist

## Data Availability

miRNA sequencing of our samples that support the findings of this study are available from the corresponding author upon request. In addition, data were released. The processed data are available from the corresponding author upon reasonable request. All original data underlying the selected data shown in the figures and supplemental figures are available from the corresponding authors upon reasonable request. Source data are provided with this paper.
