# Supplementary material for: Glioblastoma upregulates SUMOylation of hnRNP A2/B1 to eliminate the tumor suppressor miR-204-3p, accelerating angiogenesis under hypoxia
Source: Cell Death Dis. 2023 Feb 21;14(2):147. doi: 10.1038/s41419-023-05663-w (PMC9944918; doi:10.1038/s41419-023-05663-w)
Supplement: Supplementary file 1 — Supplementary Figures [file 41419_2023_5663_MOESM1_ESM.docx]

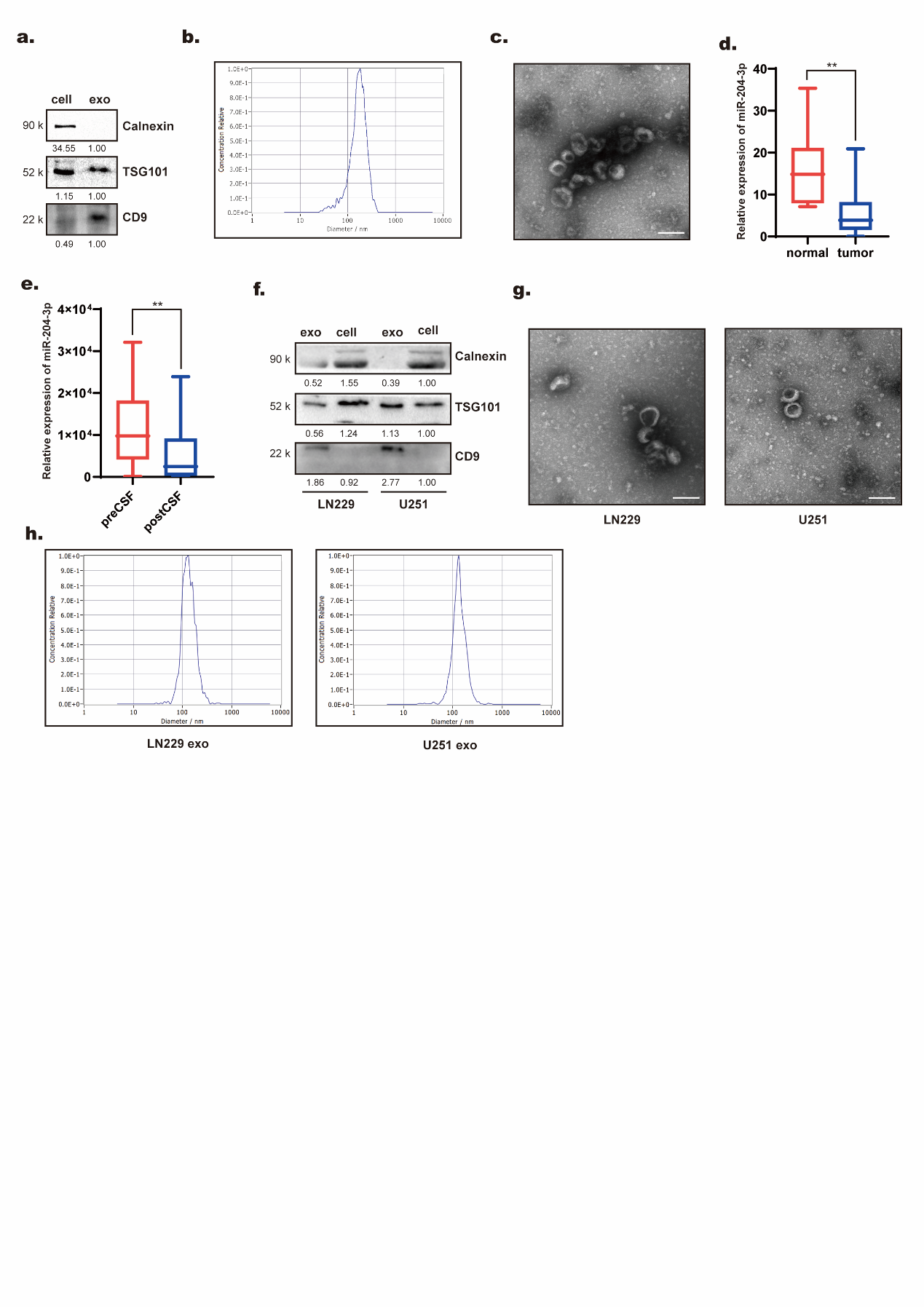


sFig.1 A Western blot analysis showed that exosome related marker proteins (TSG101, CD9) contained in CSF exosome samples while an endoplasmic reticulum-related marker protein (Calnexin) was not. B Zeta view showing particle diameter of CSF exosome samples. C Transmission electron microscopy showed the shape of CSF exosomes. Bar=200nm D Relative expression of miR-204-3p in normal tissue and glioma. E Relative expression of miR-204-3p in presurgery CSF and postsurgery CSF. F Western blot analysis showed that exosome related marker proteins (TSG101, CD9) contained in glioma cell line exosome samples while an endoplasmic reticulum-related marker protein (Calnexin) was not. G Zeta view showing particle diameter of glioma cell line exosome samples. H Transmission electron microscopy showed the shape of glioma cell line exosome. Bar=200nm


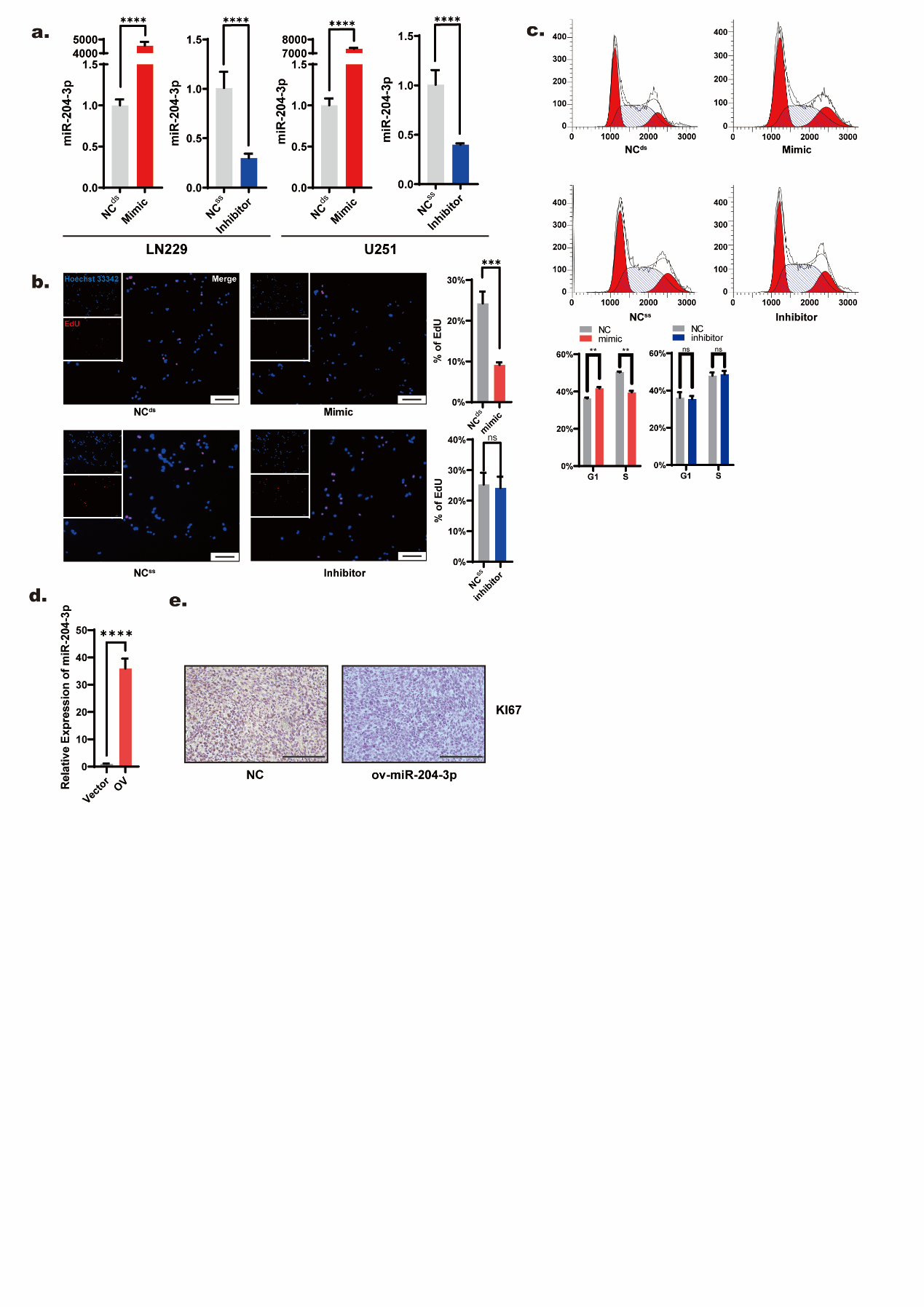


**sFig.2** A CCK8 assay showed miR-204-3p mimics suppressed the proliferation of U251 cells. B Plate colony formation assay of U251 cells after miR-204-3p mimic and inhibitor transfection. C EdU showed that the 204-3p mimic inhibited the proliferation of U251 cells. Bar=100μm. D Flow cytometry showed mimics of 204-3p caused G1/S arrest of U251.


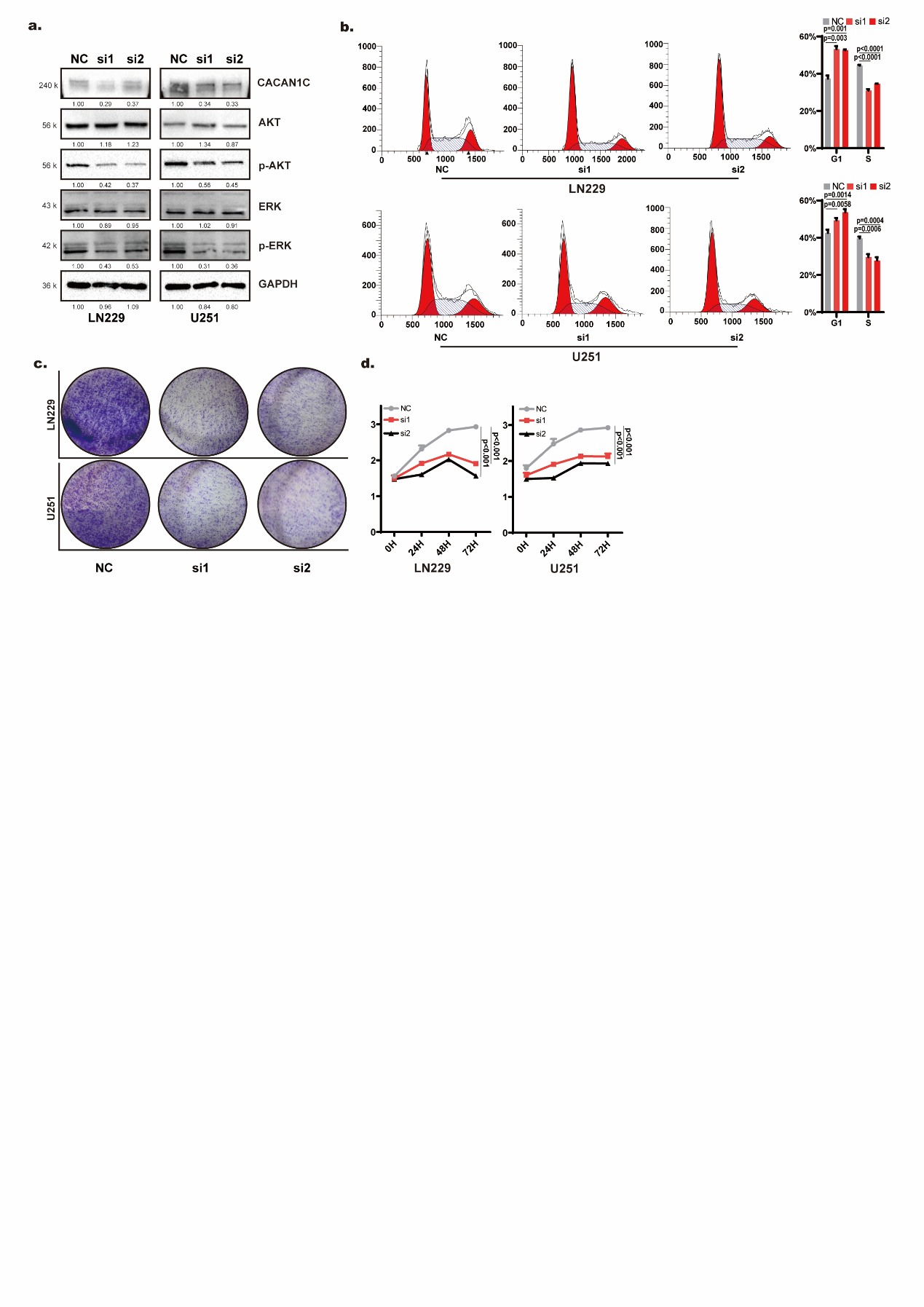


**sFig.3** A Western blot showed that knockdown of CACNAV1C with siRNA downregulated P-AKT and P-ERK but had no significant influence on either AKT or ERK. B-D Flow cytometry, plate cloning and CCK8 assays showed that CACNAV1C knockdown inhibited the proliferation of LN229 and U251 cells.


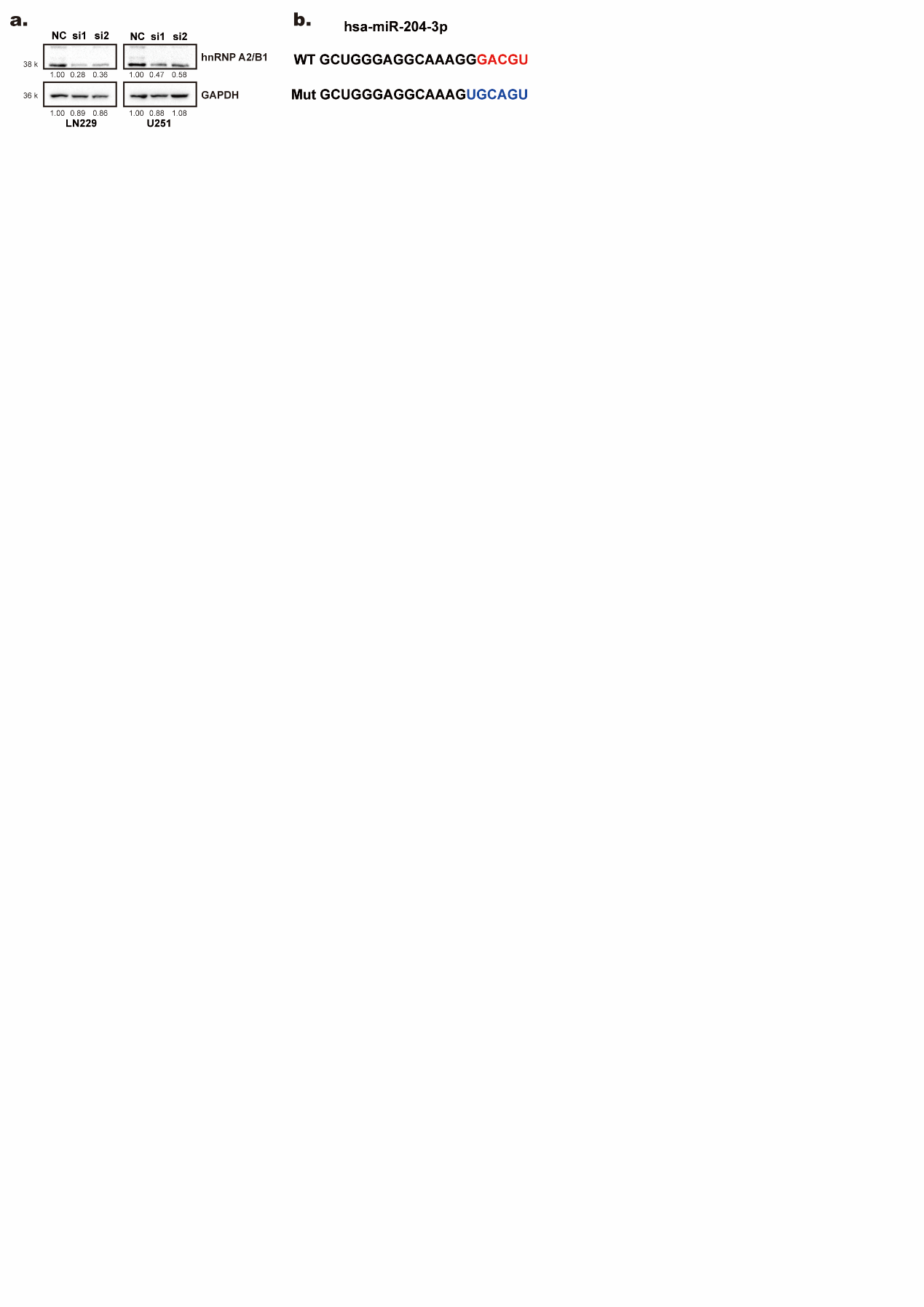


**sFig.4** A Western blot showing the knockdown efficiency of small interfering RNA of hnRNP A1/B1. B Wild (WT) and mutant (mut) sequences used in the RNA pull-down assay.


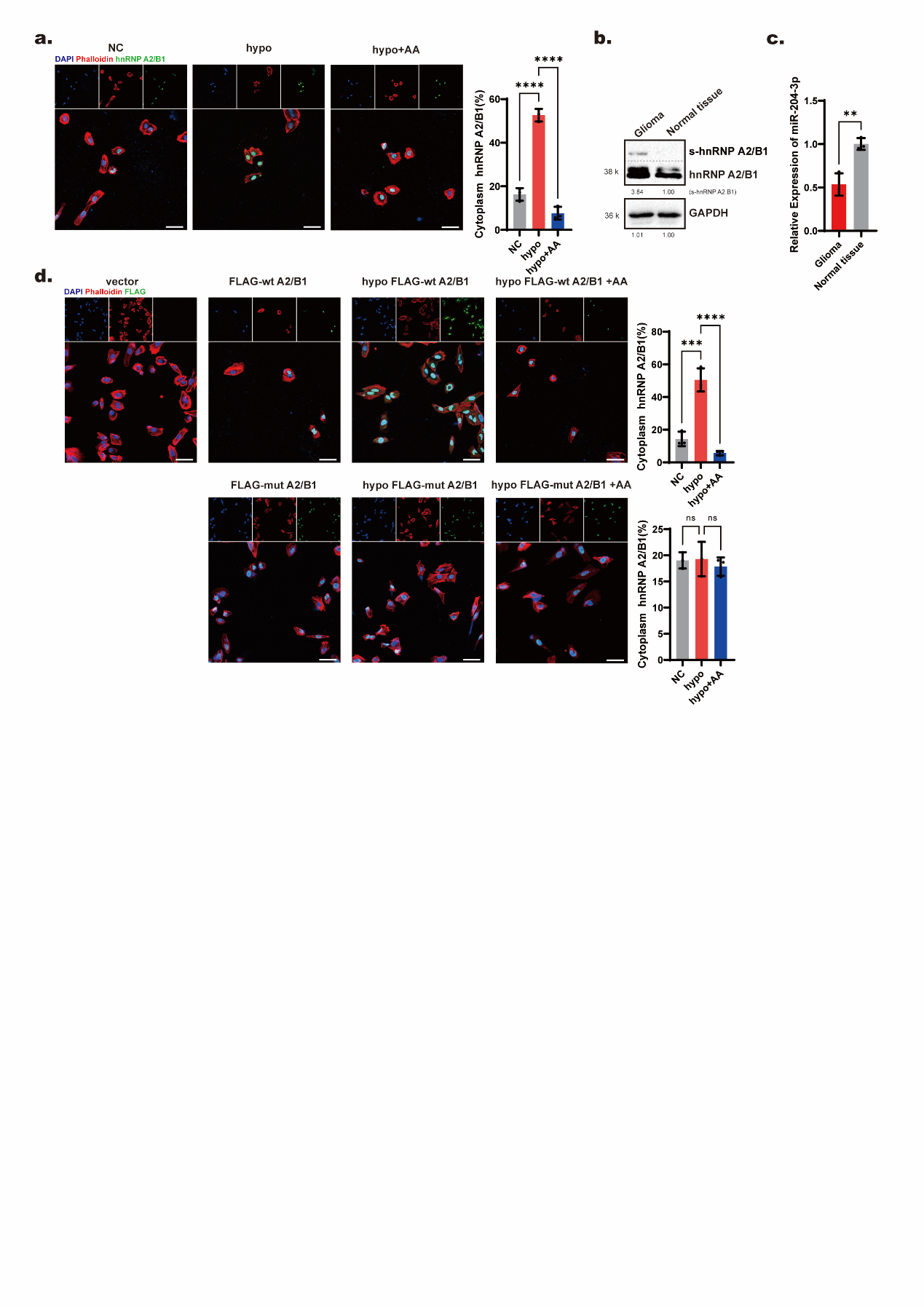


**sFig.5** A Immunofluorescence showed that the proportion of hnRNP A2/B1 increased in the cytoplasm under hypoxia, and this process could be inhibited by the SUMOylation inhibitor anacardic acid (AA) at 100 μM in LN229 cells. B Western blot analysis showed that SUMOylation level of hnRNP A2/B1 was increased in glioma tissue. C qRT-PCR showed miR-204-3p expression was down-regulated in glioma tissue. D Immunofluorescence showed that mutant hnRNP A2/B1 could not move to the cytoplasm under hypoxia in LN229 cells.


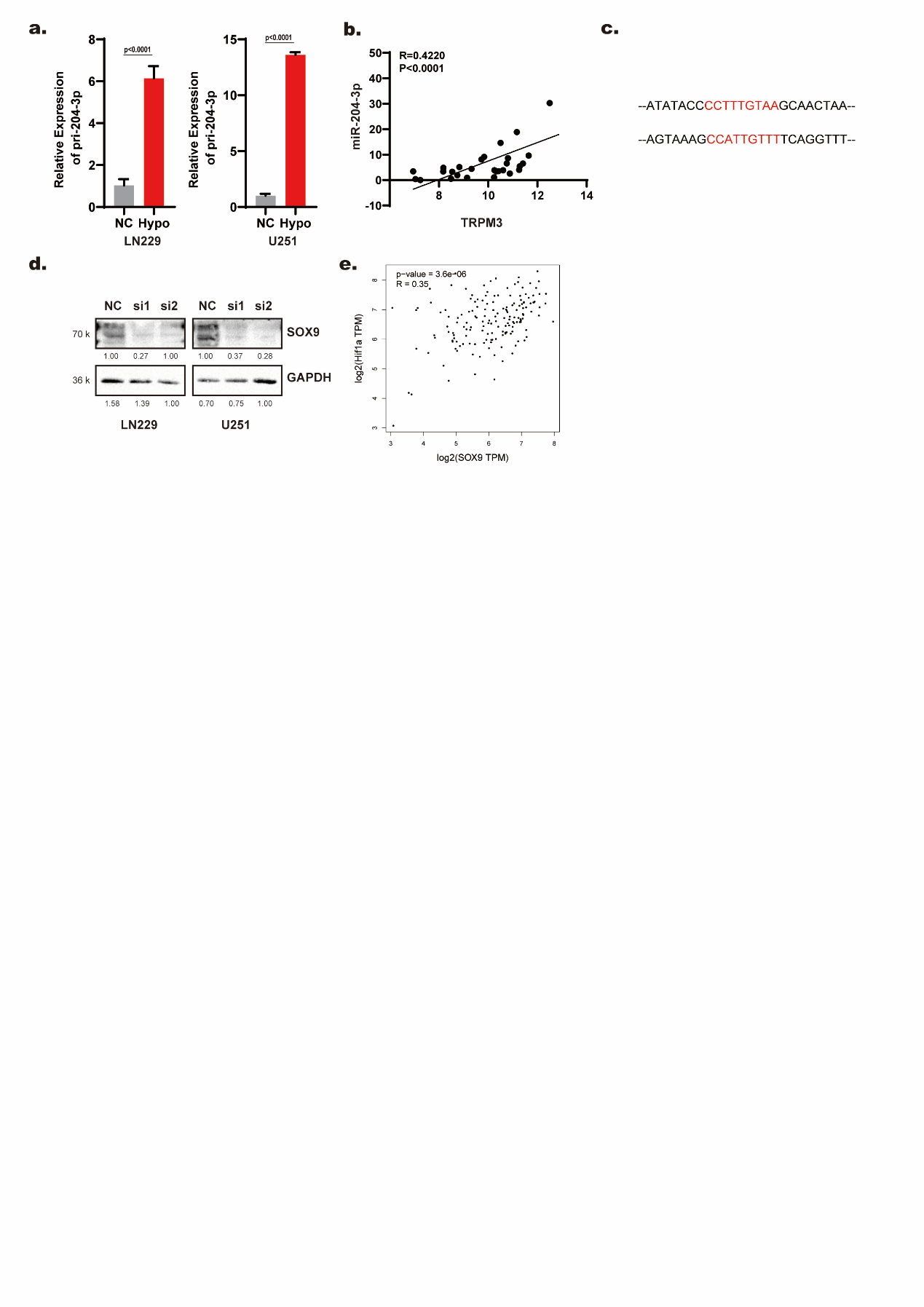
 **sFig.6** A qRT-PCR showed that pri-miR-204-3p upregulated under hypoxia. B TRPM3 was positively correlated with miR-204-3p in glioma tissue. P<0.001 R=0.422 C JASPAR predicted that SOX9 could bind to the TRPM3 promoter region. D Western blot showing the knockdown efficiency of SOX9 small interfering RNAs. E TCGA database showed that SOX9 is positively correlated with HIF1α in GBM tissue.


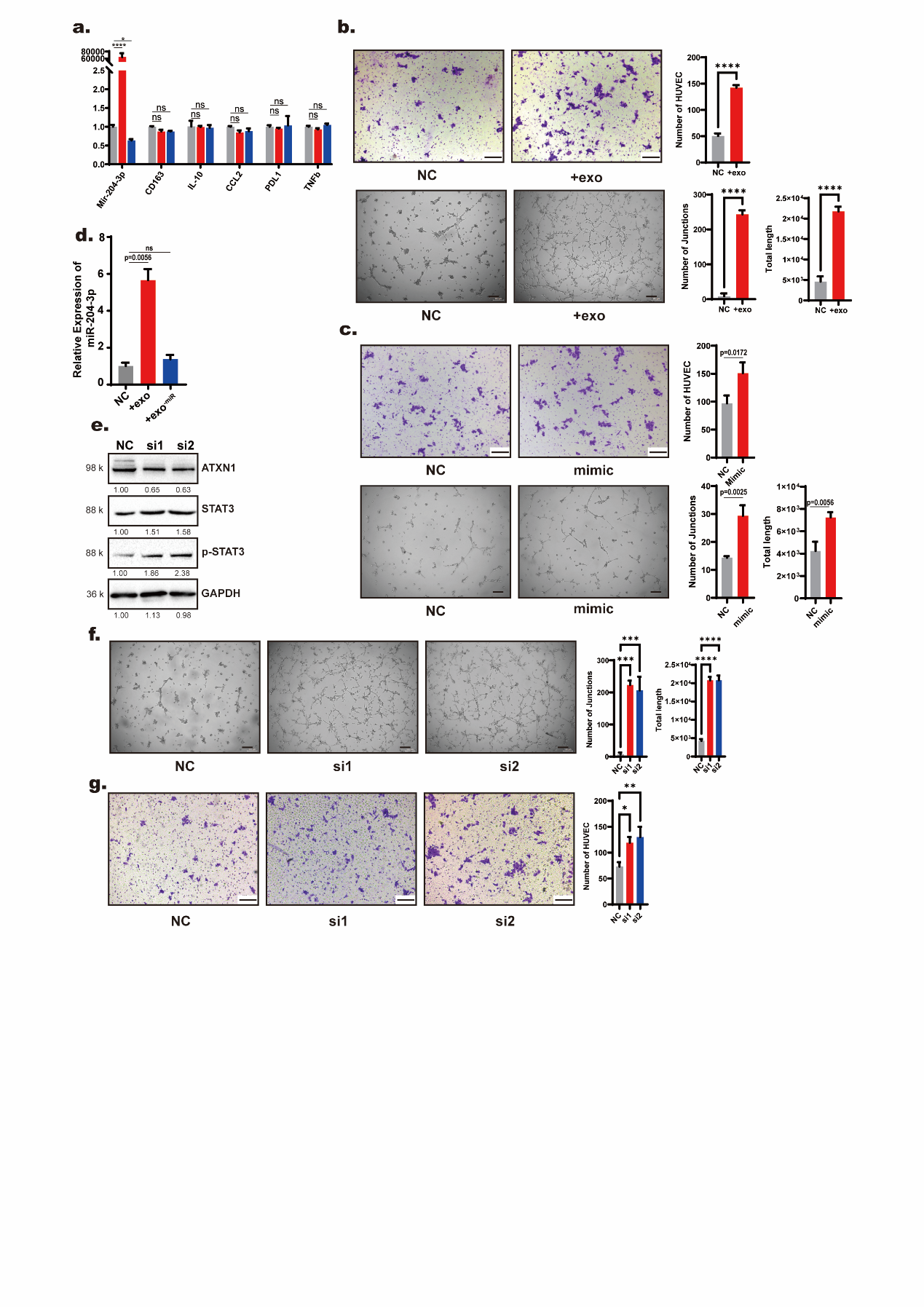


**sFig.7** A qRT-PCR showed that transfection of miR-204-3p mimics and inhibitor did not influence M2-like markers in THP1 cells. B Exosomes from GBM patients promoted angiogenesis and migration of HUVECs. Bar=100μm C Transfection of miR-204-3p promoted angiogenesis and migration of HUVECs. Bar=100μm. D Coculture with GBM exosomes increased miR-204-3p in HUVECs. E Western blot analysis showed that knockdown ATXN1 with small interfering RNA upregulated STAT3 and p-STAT3. F-G Knockdown of ATXN1 promoted angiogenesis and migration of HUVECs. Bar=100μm.
