# Supplementary figures and images for: Glioblastoma upregulates SUMOylation of hnRNP A2/B1 to eliminate the tumor suppressor miR-204-3p, accelerating angiogenesis under hypoxia
Source: Cell Death Dis. 2023 Feb 21;14(2):147. doi: 10.1038/s41419-023-05663-w (PMC9944918; doi:10.1038/s41419-023-05663-w)

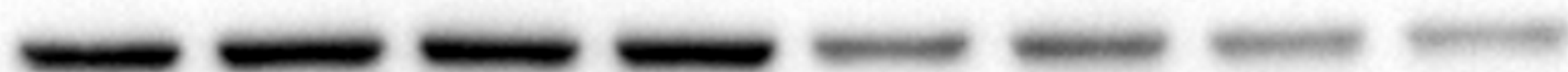

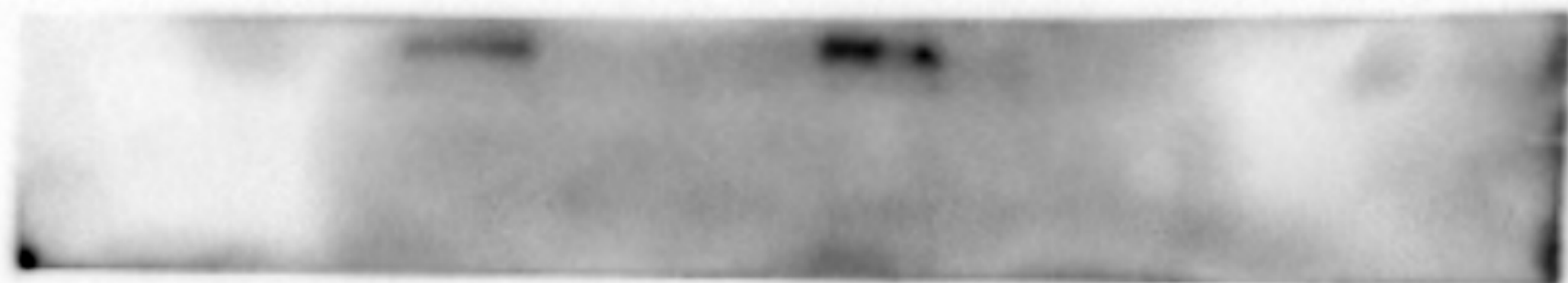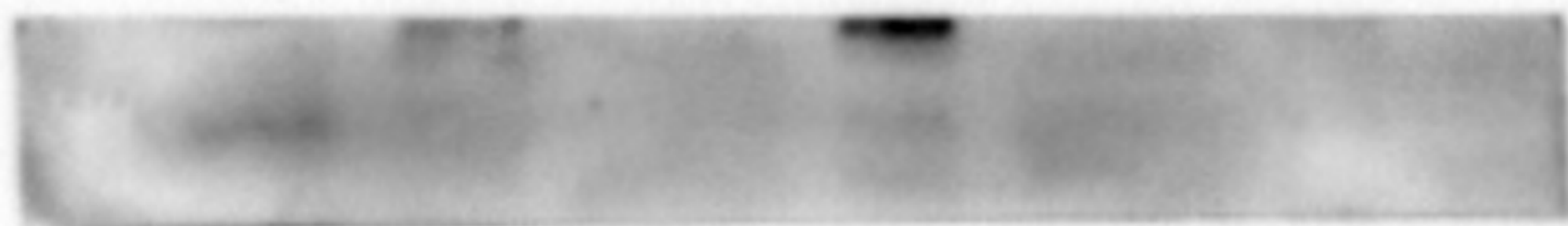

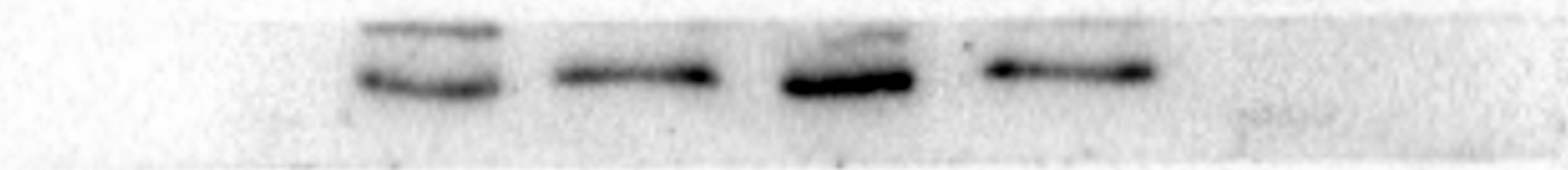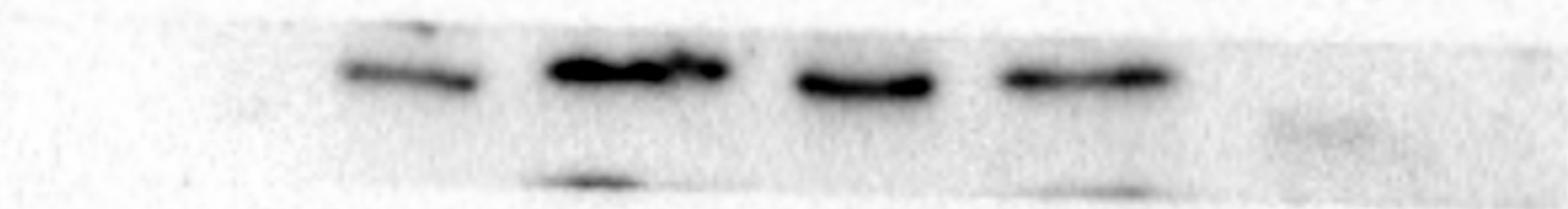

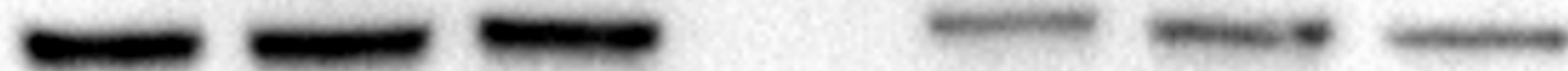

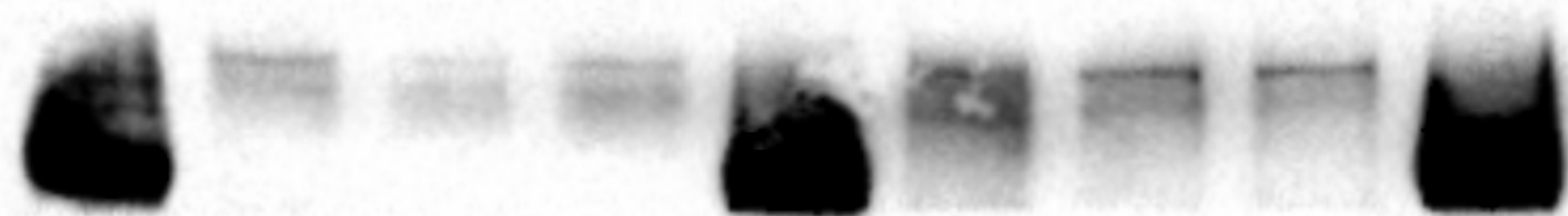

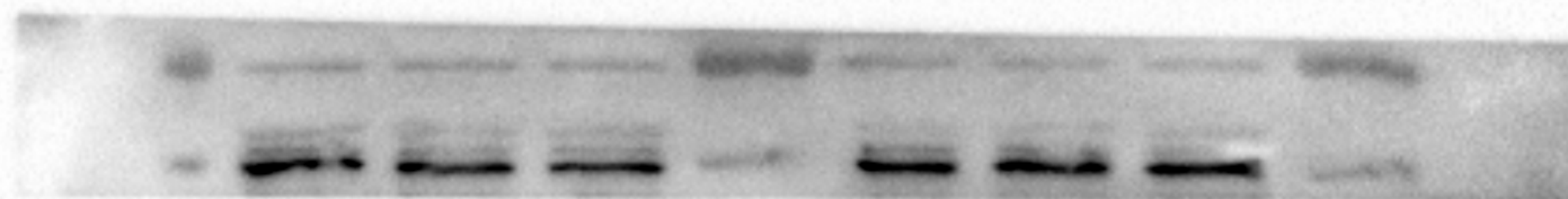

—

—

— — —

— — —

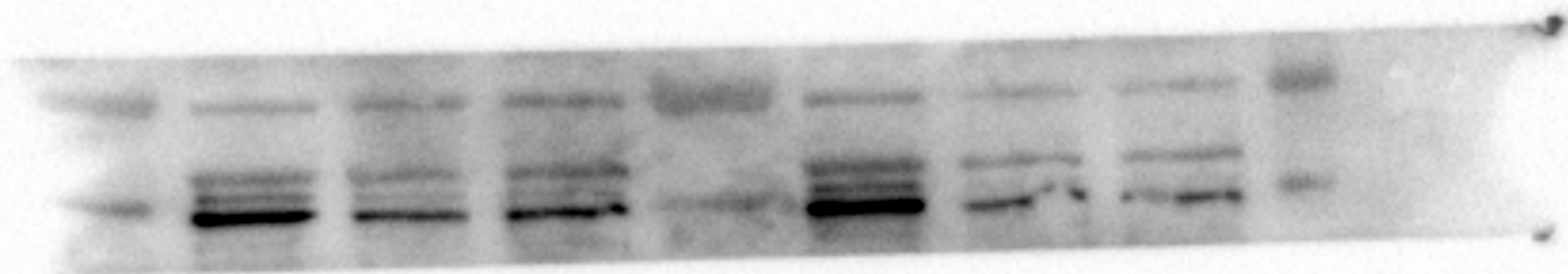

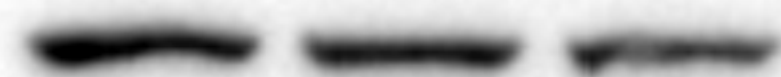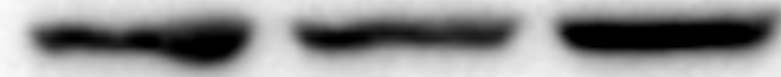

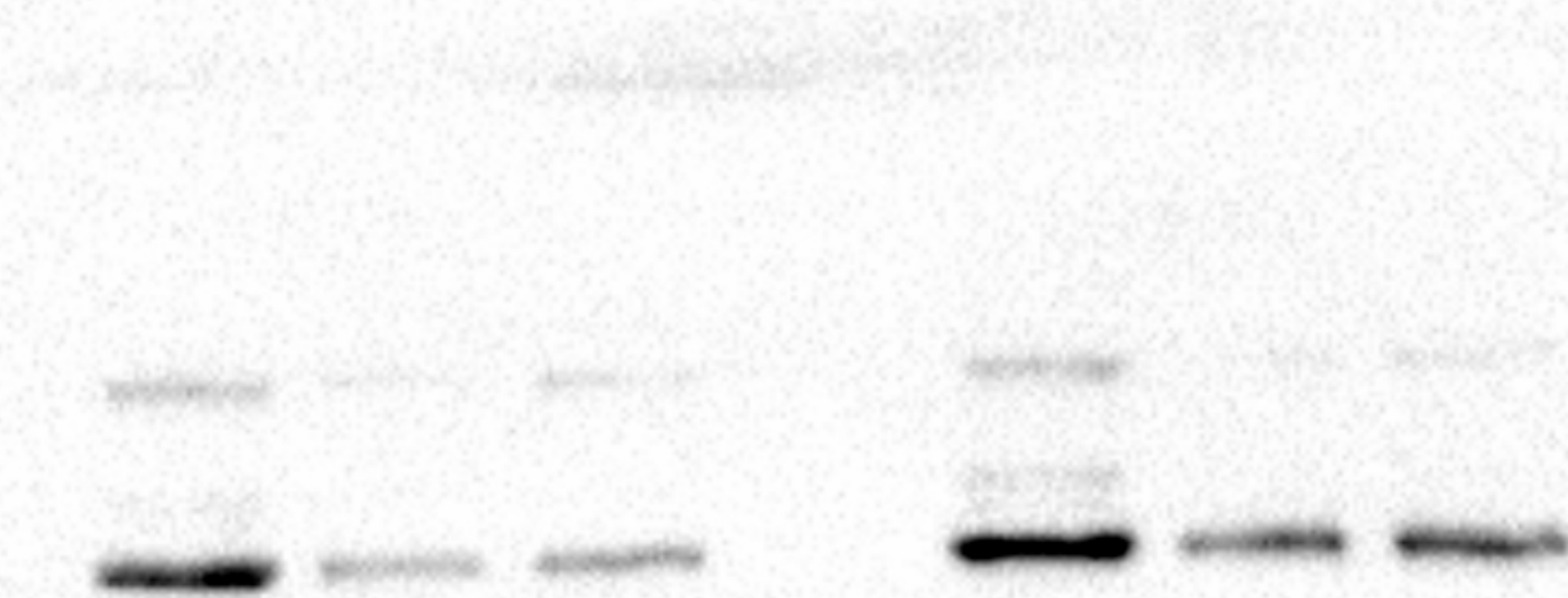

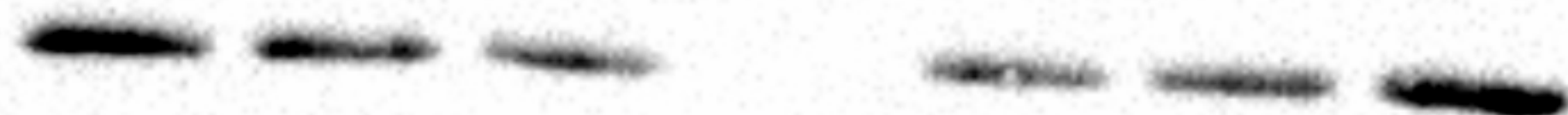

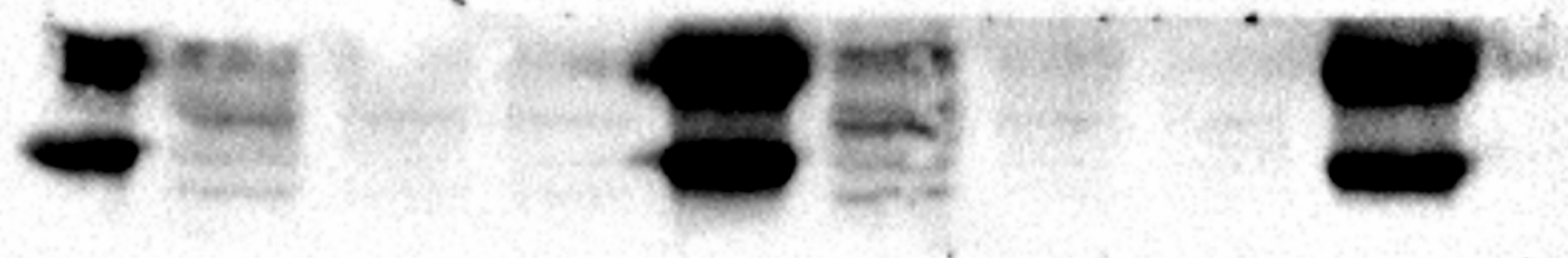

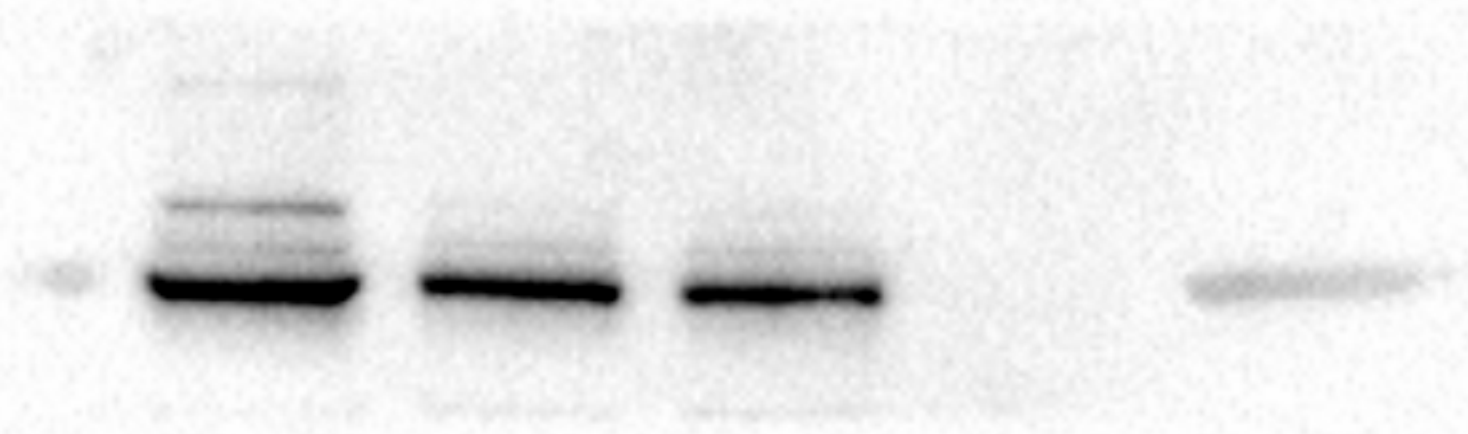

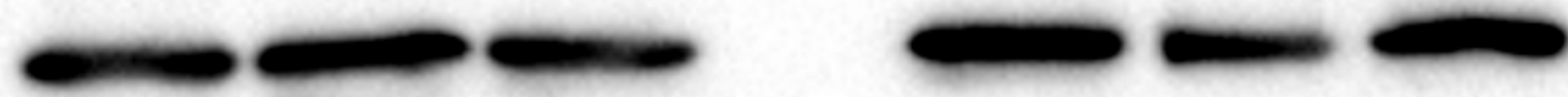

— — — — —

— — —

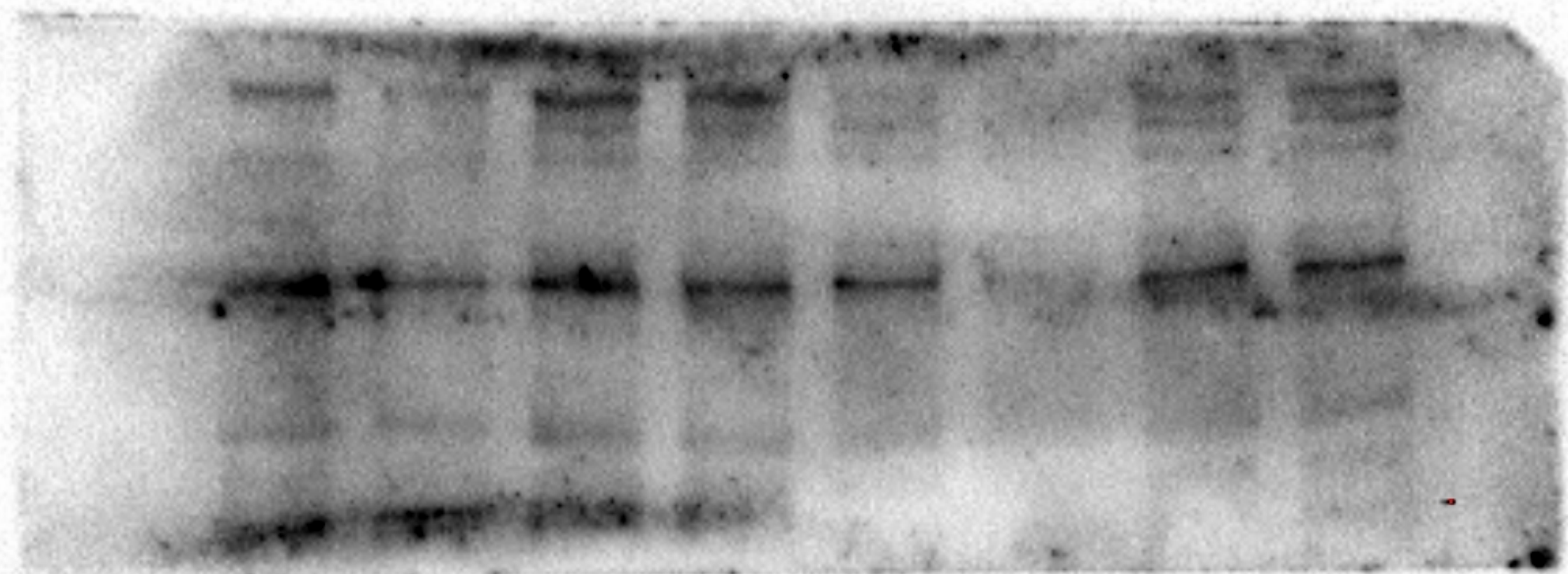

— — — — —

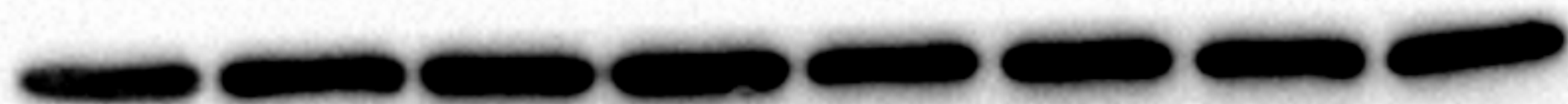

— — — — —

— — — — —

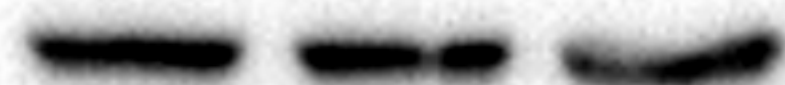

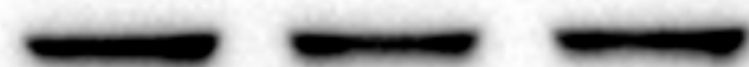

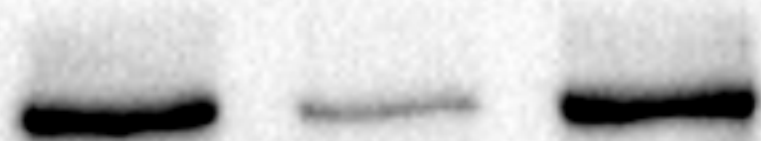

—

—

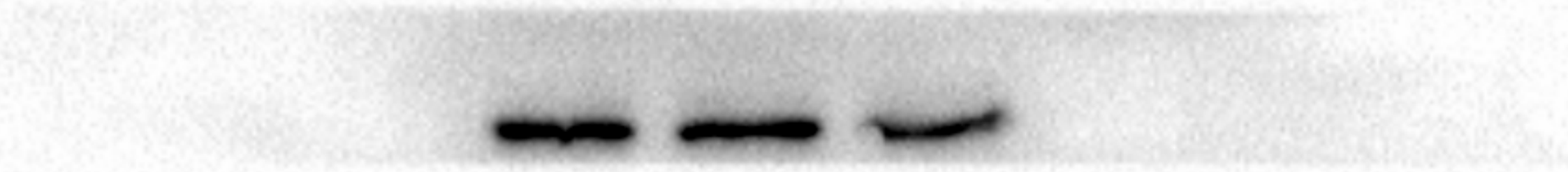

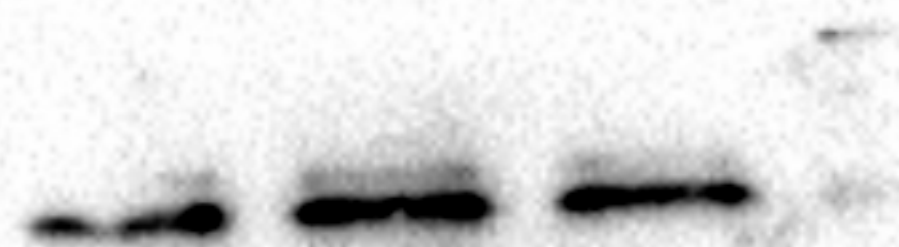

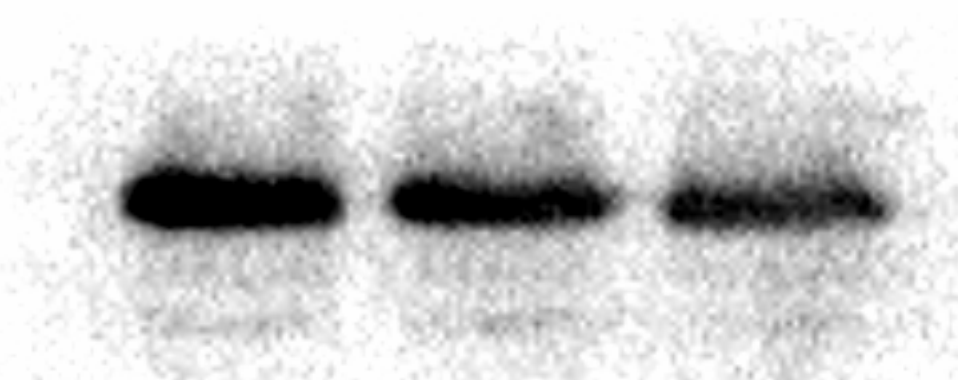

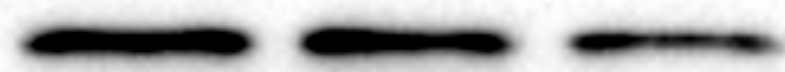

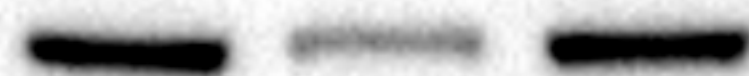

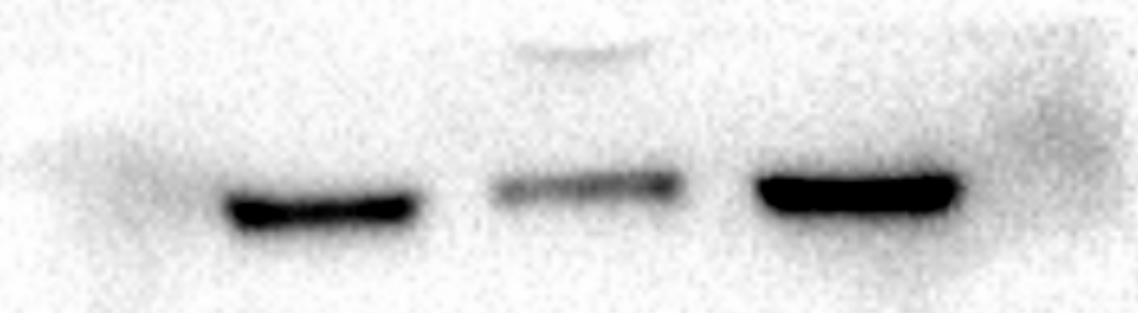

— — —

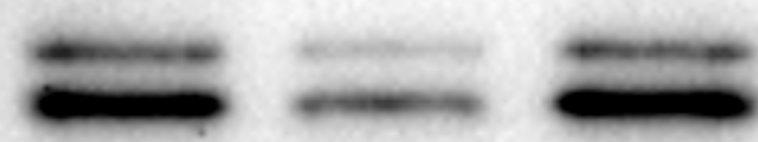

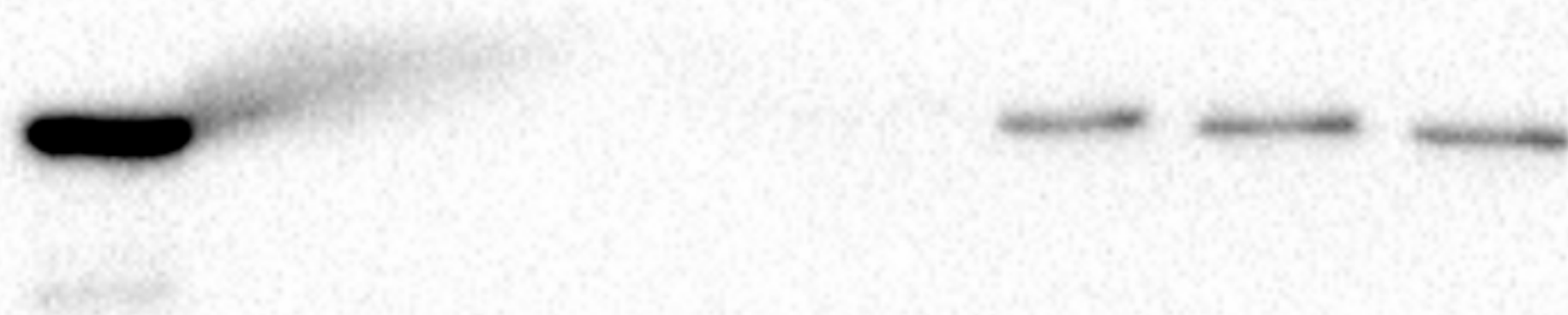

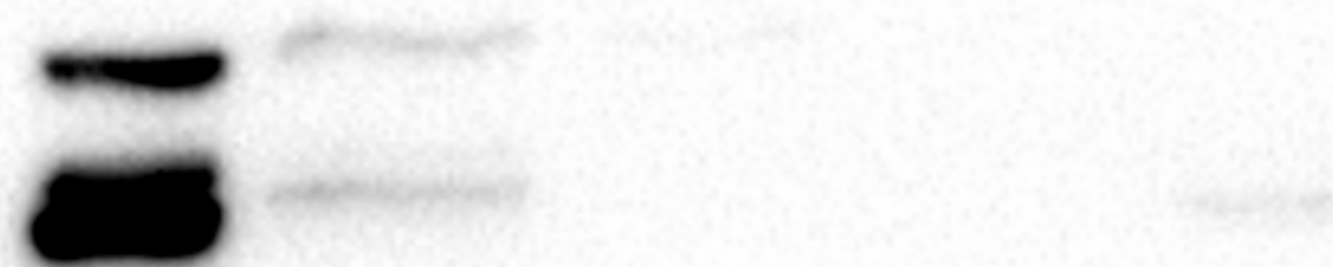

— — —

— — —

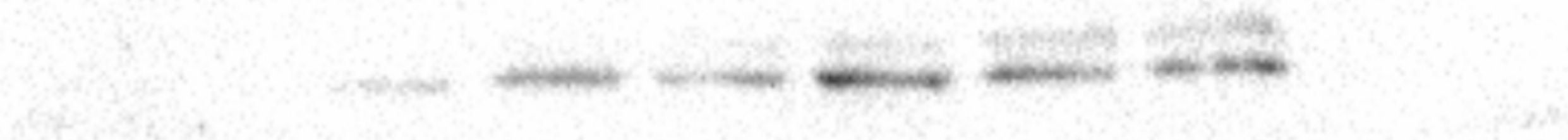

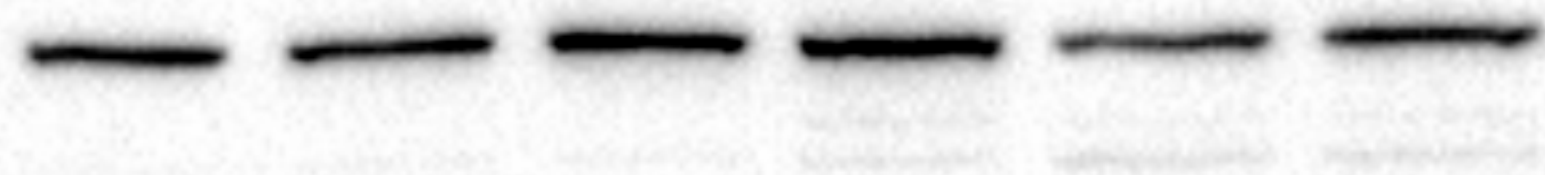

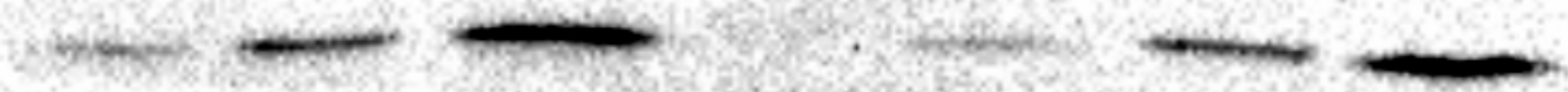

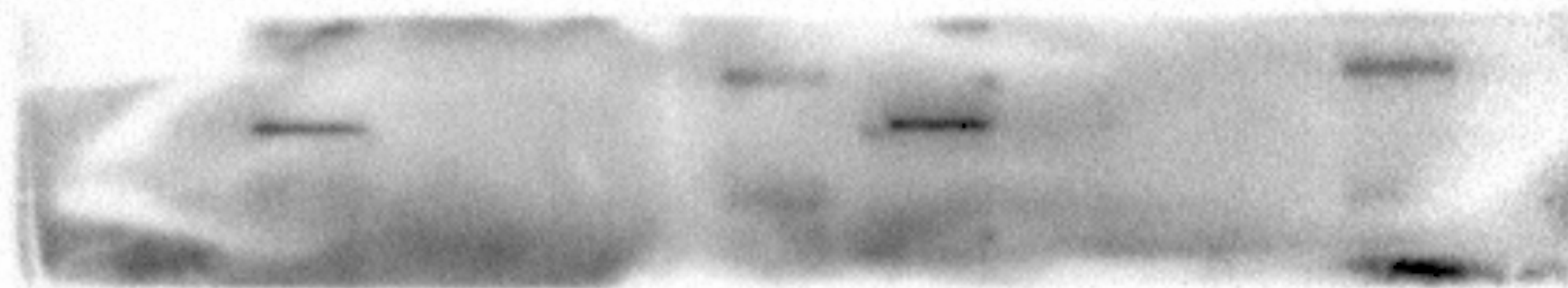

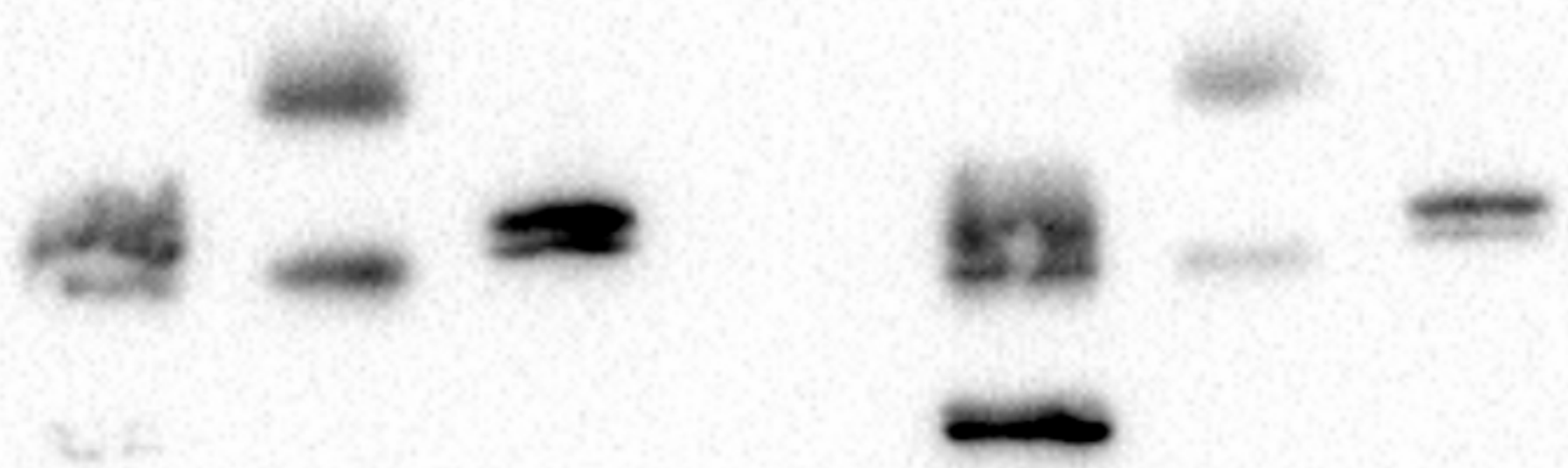

—

—

—

—

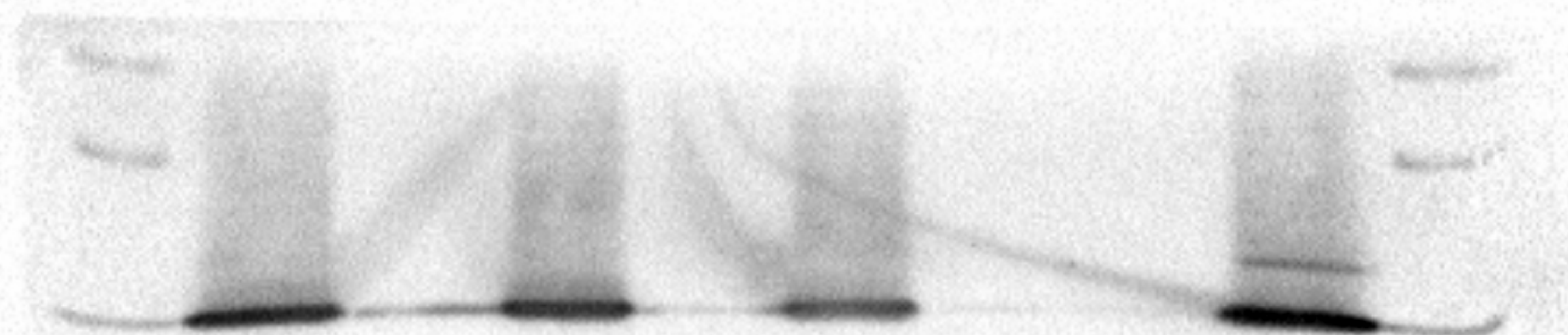

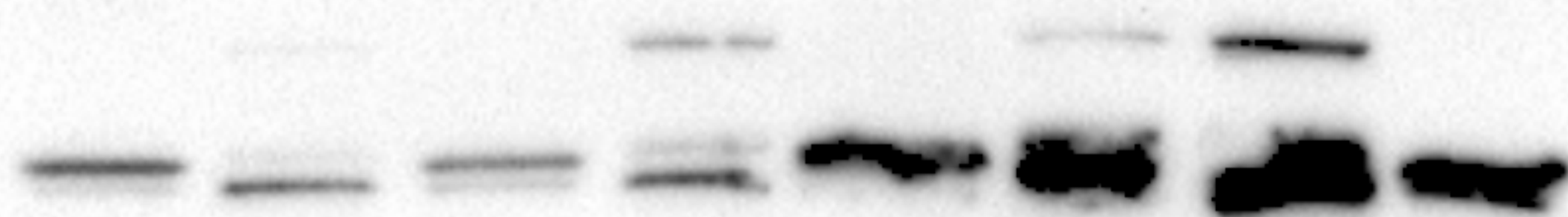

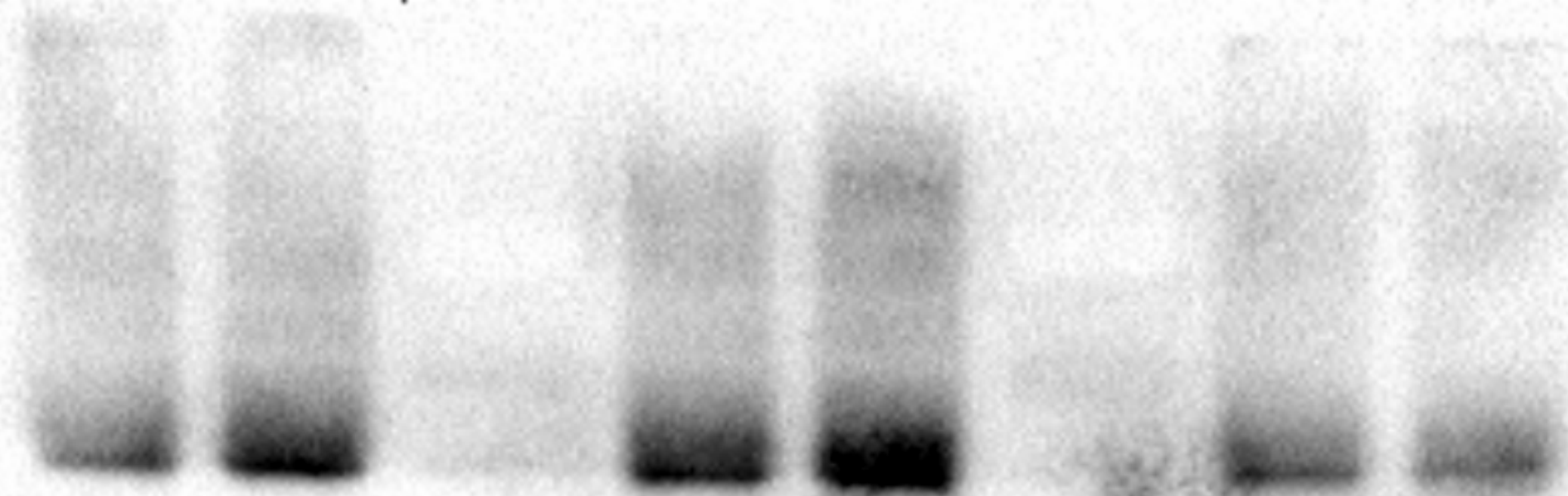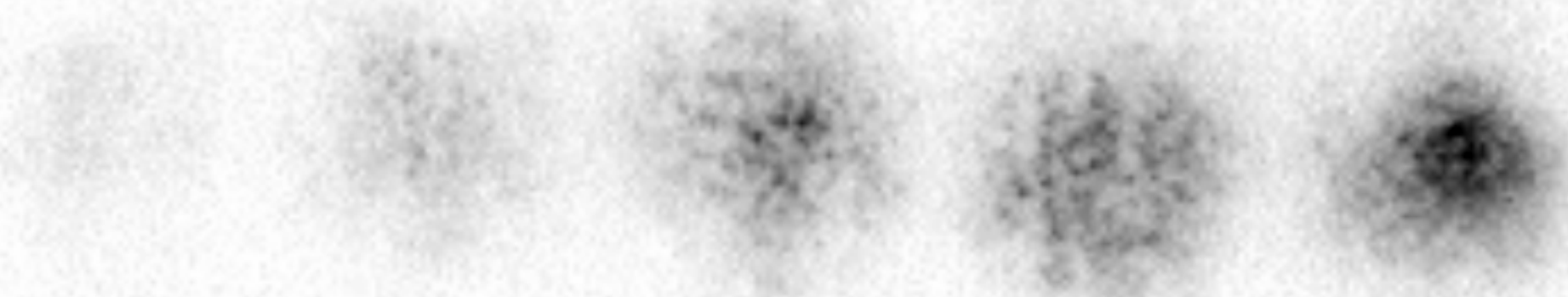

— —

— —

— —

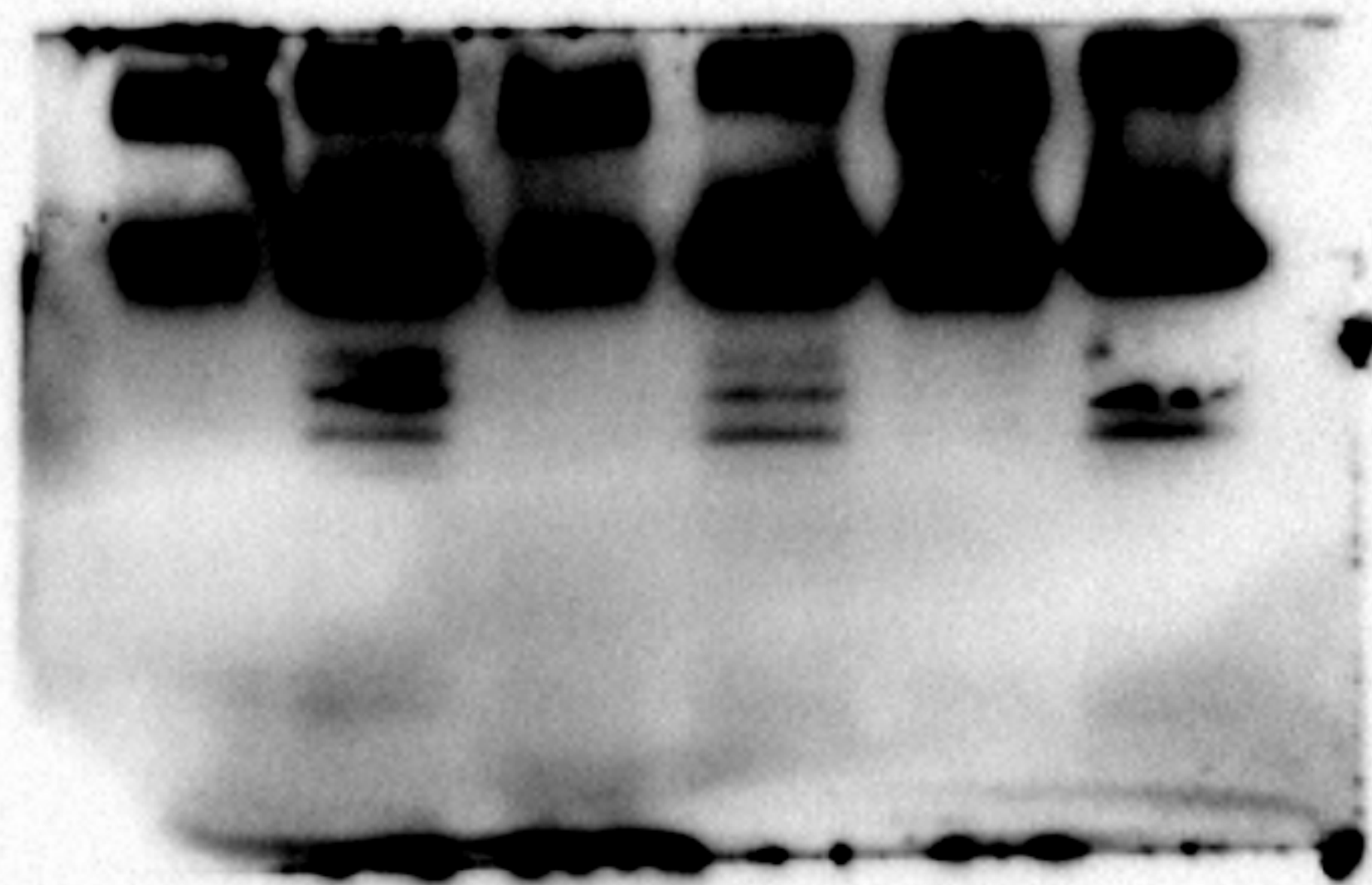

-----

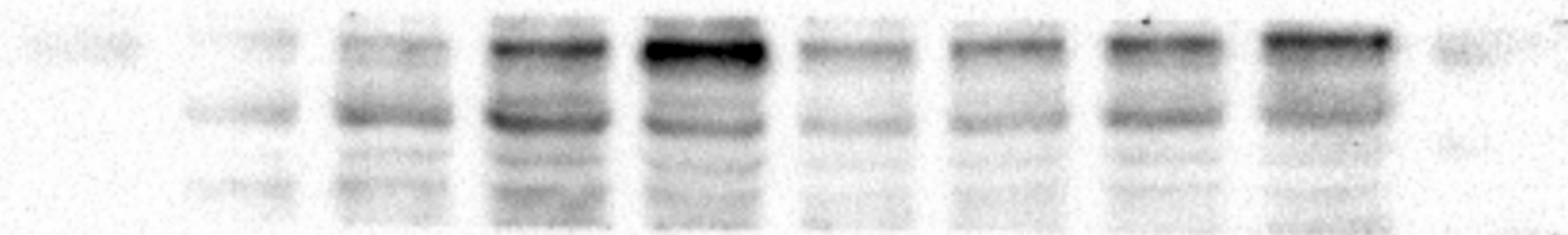

100

100

100

100

100

100

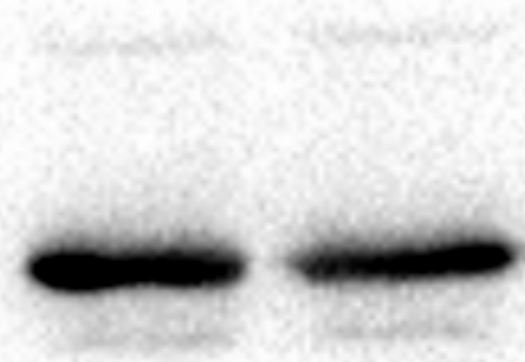

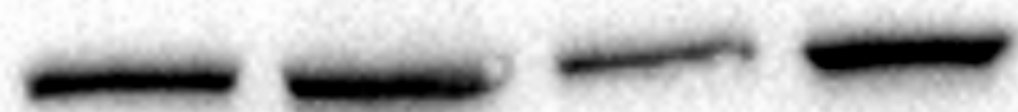

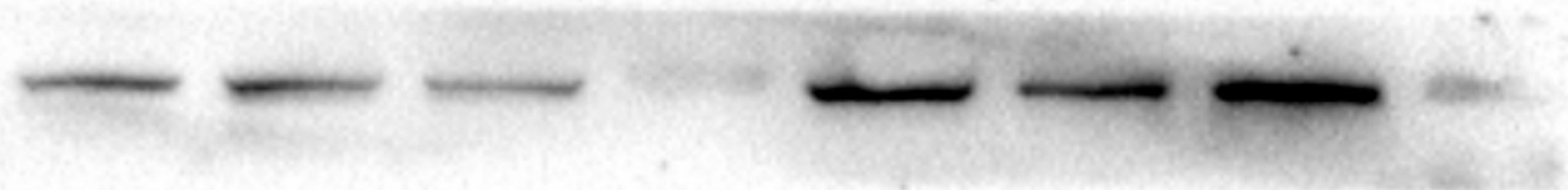

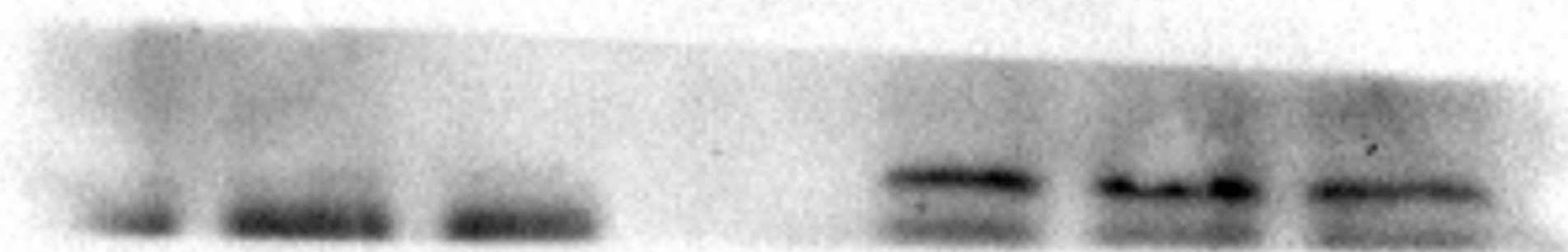

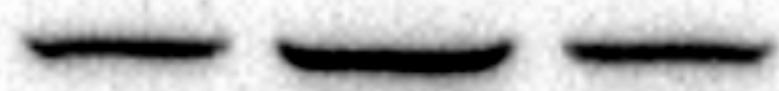

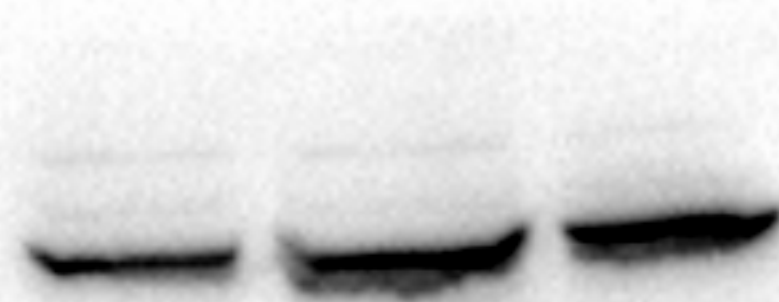

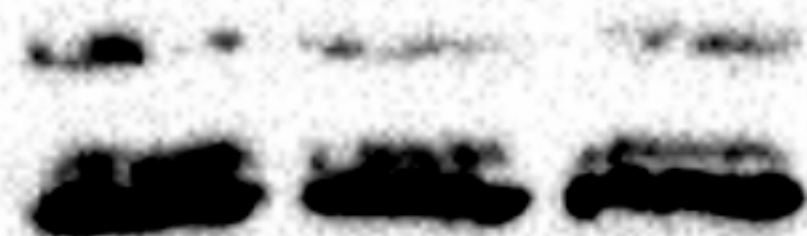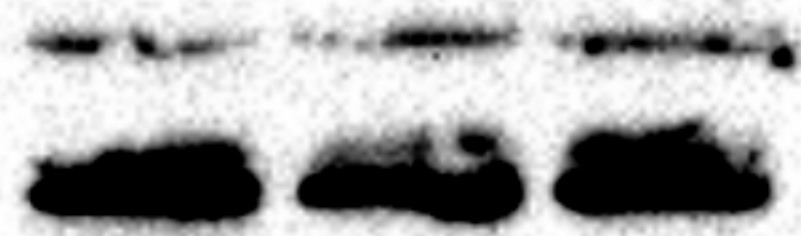

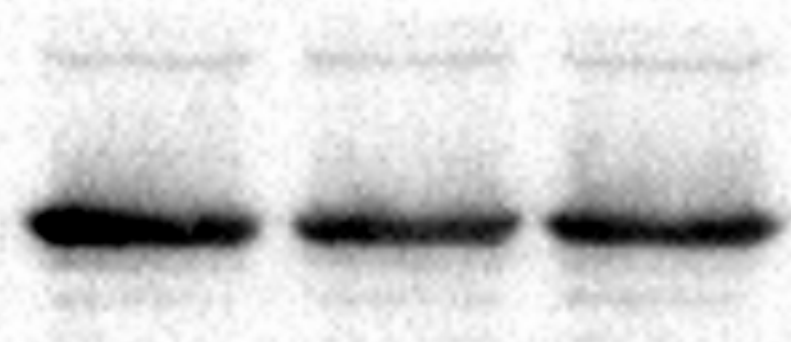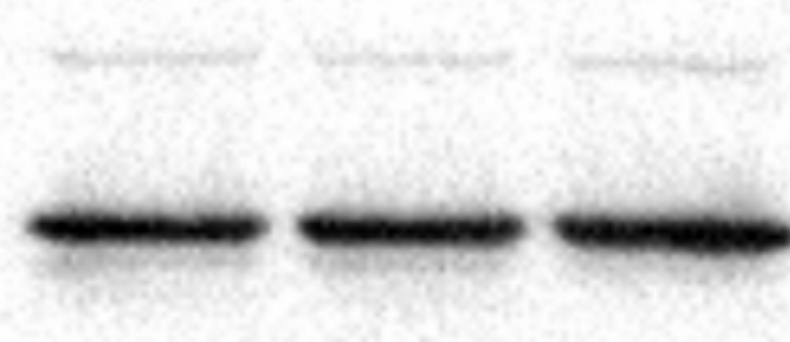

— — — — —

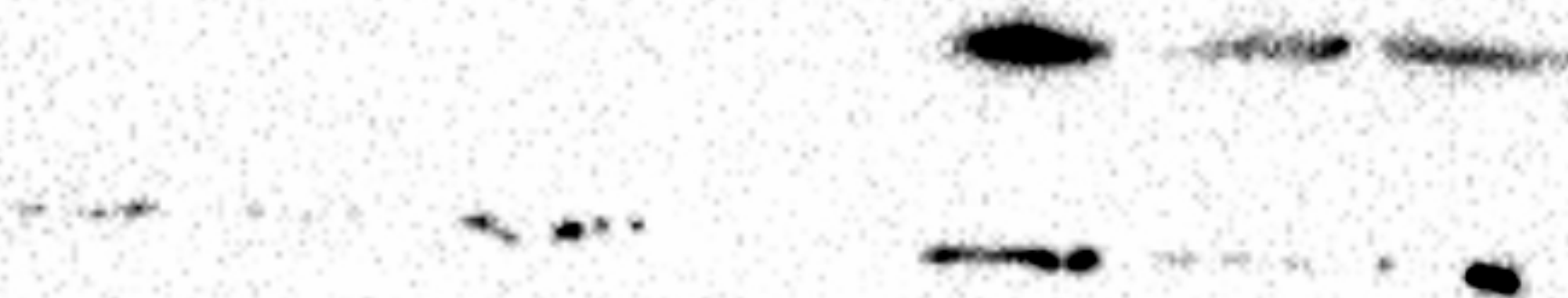

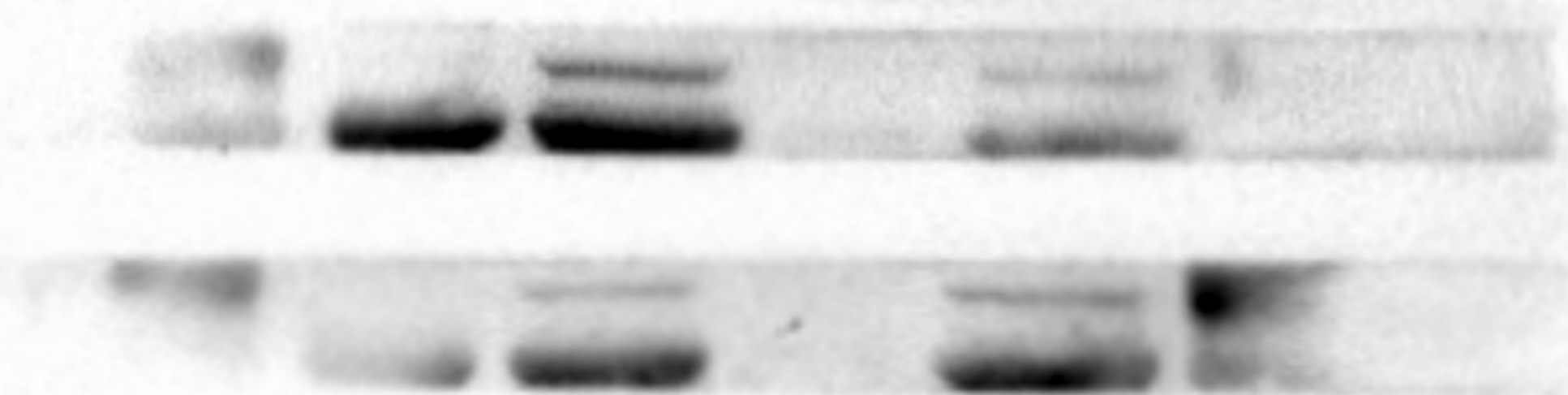

Supplement: Supplementary file 2 — Western Blot [file 41419_2023_5663_MOESM2_ESM.pdf]
